# Competing evolutionary paths in growing populations with applications to multidrug resistance

**DOI:** 10.1371/journal.pcbi.1006866

**Published:** 2019-04-15

**Authors:** Michael D. Nicholson, Tibor Antal

**Affiliations:** 1 School of Physics and Astronomy, University of Edinburgh, Edinburgh, Scotland; 2 School of Mathematics, University of Edinburgh, Edinburgh, Scotland; University of Cologne, GERMANY

## Abstract

Investigating the emergence of a particular cell type is a recurring theme in models of growing cellular populations. The evolution of resistance to therapy is a classic example. Common questions are: when does the cell type first occur, and via which sequence of steps is it most likely to emerge? For growing populations, these questions can be formulated in a general framework of branching processes spreading through a graph from a root to a target vertex. Cells have a particular fitness value on each vertex and can transition along edges at specific rates. Vertices represent cell states, say genotypes or physical locations, while possible transitions are acquiring a mutation or cell migration. We focus on the setting where cells at the root vertex have the highest fitness and transition rates are small. Simple formulas are derived for the time to reach the target vertex and for the probability that it is reached along a given path in the graph. We demonstrate our results on several scenarios relevant to the emergence of drug resistance, including: the orderings of resistance-conferring mutations in bacteria and the impact of imperfect drug penetration in cancer.

## Author summary

How long does it take for a treatment naive, growing bacterial colony to be able to survive exposure to a cocktail of antibiotics? En route to multidrug resistance, what order did the drugs become impotent in? Questions such as these that pertain to the emergence of a significant cell type in a growing population arise frequently. They are often investigated via mathematical modelling but biologically insightful results are challenging to obtain. Here we outline a general framework of a stochastically growing population spreading through a graph to study such questions and provide simple formulas as answers. The significant cell type appears upon the population reaching a target vertex. Due to their simplicity, the derived formulas are widely accessible and can be used to guide and develop intuition on a range of biological scenarios. We demonstrate this on several settings including: how a region where drugs cannot penetrate affects the emergence of resistance, and, the ordering of mutations that leads to drugs being ineffective.

## Introduction

The timing and manner in which a particular phenotype arises in a population is a central question of theoretical biology [1–13]. A typical scenario is to consider an initially monomorphic, wild type population, composed of cells that can acquire mutations, for example single site substitutions on the genome. The phenotype of interest comes to exist after a cell has accrued a specific set of mutations. The interpretations of this event are application dependent, but examples are the genesis of cancer instigated by mutations in a pair of tumour suppressor genes, or the emergence of multidrug resistance via alterations to the genes coding for the target proteins. Regardless of context, the questions of when, and how, the phenotype emerges are of significant interest.

It is commonly assumed that the population under consideration is of fixed size. However, with the aim of characterising disease progression, an increasing body of research has been developed to examine the evolutionary dynamics of a growing population. These studies have provided insights on a range of applications, including; cancer genetics [14–17], metastasis formation [18–22], drug resistance [23–26], phylogenetics [27], and the impact of poor drug penetration [28–31].

Here we continue in the same vein by considering a stochastically growing cellular population, where cells can transition in such a fashion so as to alter their, and their offsprings, reproductive capabilities. Such a transition might be due to the acquisition of a (epi)genetic alteration or migration into a new environment. As before, suppose we have a cellular state of interest, for example; a given genotype, a spatial location, or a combination of both. Will this state ever be reached? If it is, when is it reached? And by which sequence of intermediate states?

**Fig 1:**
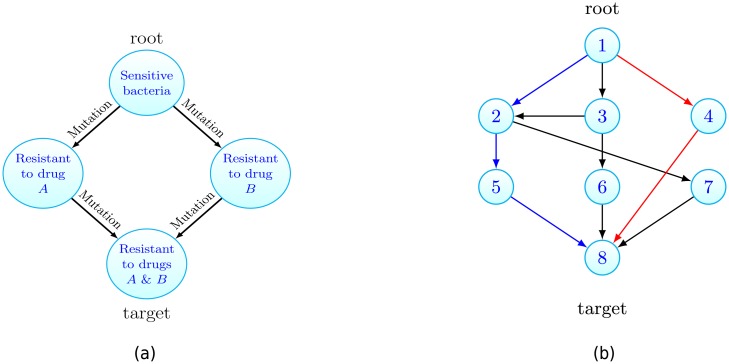
(a) Motivating example: consider a population of growing drug sensitive bacteria. Each vertex represents a cell type and cells can mutate to acquire resistance. Starting from one sensitive bacterium that can replicate, die or mutate, how long does it take to traverse the graph until multidrug resistance emerges? Is resistance to drug *A* then *B* more likely than the converse? (b) For the general case, the growing population is composed of different cell types that can be associated to vertices on a graph. Cells at vertex *x* can transition to vertex *y* if the edge (*x*, *y*) exists. Starting with cells at the root vertex, how long until the target is populated? Is the blue or red path more likely to initiate the target population? Here *N* = 8.

To make the discussion clear, let us consider an example application: the emergence of multidrug resistant bacteria. Suppose an infection begins with a single pathogenic bacterium which is sensitive to two antibiotic therapies, drug *A* and drug *B*. In the absence of either drug, the initial bacterium initiates a growing colony. Within the colony, when a cell replicates one of the daughter cells can acquire a mutation yielding resistance to either of the drugs. Here our questions are: how long does it take for multidrug resistance to emerge? En route, is resistance to drug *A* or drug *B* more likely to arise first? An ability to answer such questions is key to understanding pre-existing resistance, a common cause of therapy failure in various settings [32]. This scenario is illustrated in Fig 1a. There each vertex represents a cellular type, in this case its resistance profile. The edges represent cell transitions via mutation upon replication.

In this article we focus on the setting where the intermediate states have reduced reproductive ability (fitness) relative to the initial cells. This is primarily motivated by the commonly observed cost of resistance [33–35], whereby cellular populations that are resistant to a given drug grow more slowly than their sensitive counterparts. A second scenario where we expect a reduction in fitness is drug sensitive cells becoming exposed to toxins, which increases the cell’s rate of dying (cytotoxic) or decreases the rate of replication (cytostatic). As in many biological applications the relevant transition rates are small (some representative examples are: measurements for the point mutation rate per cell division of 10^−9^ in cancer [36] and 10^−10^ for bacteria [37], while the dissemination rate of pancreatic cancer cells from the primary tumour was estimated as 10^−7^ per cell division in [19]), we concentrate our efforts on the regime of low transition rates. Our main contribution is to provide simple, intuitive formulas that answer the questions posed in the small transition rate limit. These formulas show explicitly the contribution of the model parameters, e.g. transition rates and fitness reductions, which allow them to be probed for biological insight. This provides relationships which would be difficult to deduce from simulation alone.

We now move to detail the general framework we study, which will be seen to encompass models introduced in previous works as special cases [25, 26, 30, 38]. Our main results concerning when and how a particular cell type emerges are then presented, and we follow this by demonstrating our method on several applications.

## Results

### General framework

Our interest will always be in the emergence of a particular cell type in a growing population which is modelled as a specific form of a multitype branching process [39]. It is convenient to picture the population on a finite, simple, directed graph *G* = (*V*, *E*), see Fig 1b, where each vertex of the graph represents a cell type or state. The number of types in the system is denoted *N* and so we let *V* = {1, 2, …, *N*}. Thus *E* is a subset of the set of ordered pairs {(*i*, *j*): *i*, *j* ∈ *V*, *i* ≠ *j*}. For any given cell type there is an associated vertex in *V*. Henceforth we will refer to cells of type *x* as cells residing at vertex *x*. We will be concerned with the timing, and fashion, that vertex *N*, which we refer to as the target, is populated, when we initiate with cells at vertex 1, which we denote the root. In the example of multidrug resistance, illustrated in Fig 1a, the cells at the root are sensitive bacteria while the target population is resistant to both drugs.

Let (*x*) represent a cell at vertex *x* and ∅ symbolise a dead cell. Then, with all cells behaving independently, our cell level dynamics can be graphically represented as:

$$\begin{array}{c} \left(x\right)\to \{\begin{array}{cc} (x),(x)\; & \text{at}\;\text{rate}\;\alpha (x)\\ \emptyset \; & \text{at}\;\text{rate}\;\beta (x)\\ (x),(y)\; & \text{at}\;\text{rate}\;\nu (x,y)\;\text{if}\;(x,y)\in E. \end{array} \end{array}$$

That is: cells divide at rate *α*(*x*), die at rate *β*(*x*), and transition to a cell at vertex *y* at rate *ν*(*x*, *y*) if the edge (*x*, *y*) exists. We will denote the fitness (growth rate) of cells at vertex *x* by λ(*x*) = *α*(*x*) − *β*(*x*), the difference of division and death rates of vertex *x* cells. The parameters associated with the vertex 1 population feature prominently and so for convenience we let *α* = *α*(1), *β* = *β*(1) and λ = λ(1). As mentioned above, in this article we focus on the setting where the vertex 1 population has the largest fitness, which is also positive. That is, we assume that λ > 0 and for 2 ≤ *x* ≤ *N* − 1, λ(*x*) < λ. We do not specify the fitness of the target population (cells at vertex *N*).

A common variant when modeling transitions (or mutations) is to specify that cells of type *x* divide at rate *α*′(*x*), and then with probability *ν*′(*x*, *y*) a transition occurs to vertex *y*. For a fixed value of parameters, this formulation of transitions is equivalent to that given above upon letting *ν*(*x*, *y*) = *α*′(*x*)*ν*′(*x*, *y*), *α*(*x*) = *α*′(*x*)(1 − ∑_*y*:(*x*,*y*)∈*E*_ *ν*′(*x*, *y*)). Also in the model as currently stated, transitioned cells have deterministically fixed new division and death rates. However including a finite distribution of fitness effects, when a transitioned cell is assigned random division and death rates, is actually covered by our model as given thus far. We discuss this point further in the Extensions section.

At a population level, the number of cells at vertex *x* at time *t* will be denoted *Z*_*x*_(*t*). We shall always assume that the system is initiated with only vertex 1 cells, at a quantity *z*, that is *Z*_*x*_(0) = *zδ*_*x*,1_ with the Kronecker delta function *δ*_*x*,*y*_.

We have two primary questions. The first is, having initiated the system with *z* vertex 1 cells, how long does it take for the population at the target vertex to arise? That is we concern ourself with the distribution of the target hitting time, defined as

$$\begin{array}{c} T=\inf\{t\geq 0:\;Z_{N}\left(t\right)>0\}. \end{array}$$ (1)

Now assuming that the target vertex is populated by a founding cell, we ask from which path through the graph *G* did this founding cell come? This gives rise to a distribution over the set of paths (or walks) from the root vertex to the target vertex, which we aim to characterise. This second question is more precisely formulated in the Path distribution section. Throughout we will assume that we wait for the first cell at vertex *N* to arise, however it may be that the population founded by that initial cell goes extinct. If instead one wishes to wait for the first ‘successful’ cell, that is the first cell at vertex *N* to exist whose progeny survives, then all the results presented below hold so long as the mapping *ν*(*x*, *N*) ↦ *ν*(*x*, *N*)λ(*N*)/*α*(*N*) for all target adjacent vertices *x*, is applied.

Our results are most clearly understood when *G* is acyclic, which amounts to excluding reverse transitions. Therefore in the following presentation this will be assumed. The setting when this assumption is relaxed will be discussed in the Extensions section.

Consider any path from the root to the target vertex. Intuitively, if the transition rates encountered along this path are larger, the target population will be seeded from this path quicker. Conversely, the smaller the growth rates along this path are, the slower the target will be reached. It will transpire that this intuition is indeed correct. The key quantities that show how the time to the target, and which path is taken, depend on these competing factors will be seen to be the path weights, which we now introduce.

Let the set of paths between the root and the target be denoted $P_{1,N}$. Any $p\in P_{1,N}$, will be a sequence *p* = (*p*_1_, *p*_2_, …, *p*_*l*_, *p*_*l*+1_), where each 1 ≤ *p*_*i*_ ≤ *N*, *l* is the path length (the number of edges in *p*), all *p*_*i*_ are distinct (as presently *G* is acyclic), and *p*_1_ = 1, *p*_*l*+1_ = *N*. Along the path *p*, let us call the difference between the growth rate at the root and the vertices encountered the fitness costs. Then we define the weight of path *p* as

$$\begin{array}{c} w\left(p\right)=\nu \left(p_{1},p_{2}\right)\prod_{i=2}^{l}\frac{\nu (p_{i},p_{i+1})}{\lambda -\lambda (p_{i})}, \end{array}$$ (2)

that is the product of the transition rates along the path divided by the fitness costs along the path. Throughout the empty product is set to 1. Further, we let the total weight of the target be

$$\begin{array}{c} \phi_{N}=\sum_{p\in P_{1,N}}w\left(p\right). \end{array}$$ (3)

**Fig 2:**
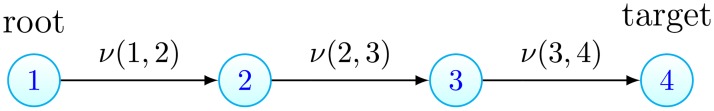
A path graph of length 3, with *N* = 4.

We will take the case of a path graph as a running example. A path graph, as illustrated in Fig 2, is the scenario in which the only edges are (*i*, *i* + 1), for 1 ≤ *i* ≤ *N* − 1. There is only one path *p* between the root and the target and so in this case, $\phi_{N}=w\left(p\right)=\nu \left(1,2\right)\prod_{i=2}^{N-1}\frac{\nu (i,i+1)}{\lambda -\lambda (i)}$.

### Time until target vertex is populated

How long do we wait until the target vertex is populated? We answer this question by characterising the distribution of *T*, defined in (1), when the target seeding transition rates (the transition rates associated with edges into the target vertex) are small. The key tool required is the long-time population numbers at the initial and intermediate vertices, which is discussed further in the Materials and Methods section. Using this we are able to prove the following theorem regarding the target hitting time, whose proof can be found in S1 Appendix (see Sections S3 and S4).

**Theorem 1**. *As the target seeding transition rates tend to 0, we have*

$$\begin{array}{c} P\left(T-\mu >t\right)\to (\frac{\lambda /\alpha }{1+e^{\lambda t}}+\beta /\alpha {)}^{z} \end{array}$$ (4)

*where*

$$\begin{array}{c} \mu =\frac{1}{\lambda }\text{log}\frac{\lambda^{2}}{\alpha \phi_{N}}. \end{array}$$

Heuristically, the time until the target is populated is approximately *μ* plus some noise, where the distribution of the noise is given by the right hand side of (4). Note that this distribution depends only on parameters associated with vertex 1 cells, while all other parameters of the system are bundled into *μ*. For practical purposes the theorem yields the following approximation

$$\begin{array}{c} P\left(T>t\right)\approx (\frac{\lambda /\alpha }{1+e^{\lambda t}\phi_{N}\alpha /\lambda^{2}}+\beta /\alpha {)}^{z} \end{array}$$ (5)

**Fig 3:**
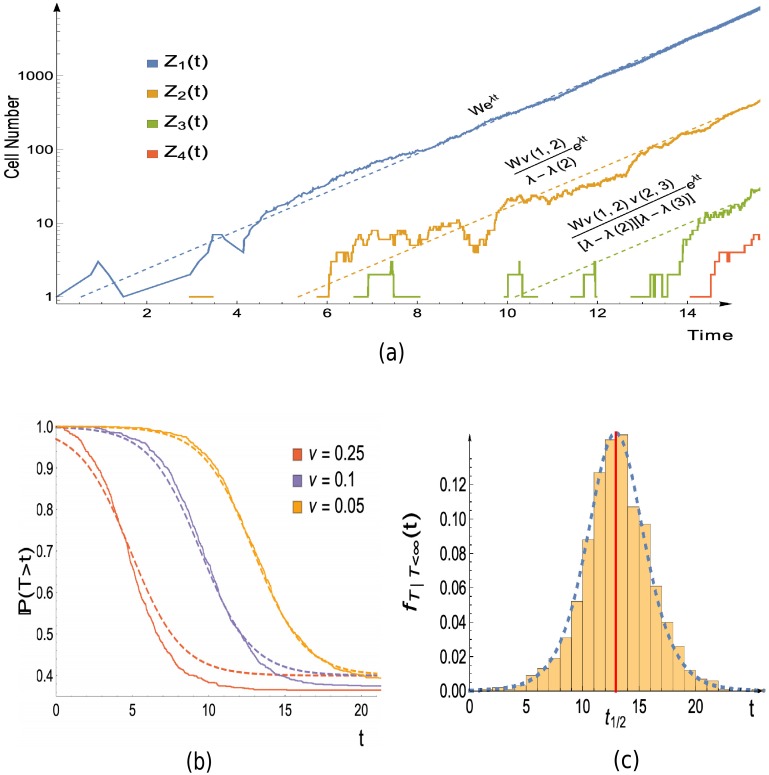
Simulation versus theory in the case of a path graph with 4 vertices, as in Fig 2. (a) Joined lines show a single realisation of the population growth obtained via simulation. Dashed lines are the asymptotic growth of the populations, which follows the functions given immediately above the dashed lines. These functions are a consequence of Theorem 3, which can be found in the Materials and Methods section. *W* is a random variable whose value will differ between simulations. Its value here is estimated by the realised value of $e^{-\lambda t_{f}}Z_{1}\left(t_{f}\right)$, with *t*_*f*_ = 14.5. (b) The distribution of the hitting time for vertex 4: Joined lines show the empirical distribution obtained from 1000 realisations, dashed lines display the theoretical approximation (5). The error in the approximation decreases as *ν* → 0, but still shows good agreement for moderate values of *ν*. (c) Probability density function for hitting time of vertex 4, conditioned that it is finite: bars display normalised histogram constructed from 1000 realisations, while dashed line shows approximation which follows from differentiating the conditional variant of (5) (given in Eq. S79 of S1 Appendix). The approximate median time for the type 4 population to arise, as given (9), is indicated by the red vertical line. Parameters: *α* = *α*(2) = *α*(3) = *α*(4) = 1, *β* = 0.4, *β*(2) = *β*(3) = 1.3, *β*(4) = 0.6, *z* = 1; for (a) and (c), *ν*(1, 2) = *ν*(2, 3) = *ν*(3, 4) = 0.05; for (b), legend displays value of *ν* = *ν*(1, 2) = *ν*(2, 3) = *ν*(3, 4) used.

This approximation will be valid if the target seeding transition rates are small. We do not provide estimates on the approximation error, however simulations (for example see Fig 3b) demonstrate this approximation holds even for only moderately small transition rates.

Notice that it is possible that the target is never populated (*T* = ∞). In S1 Appendix (Proposition S1) we show that if the vertex 1 population survives forever then the target hitting time is finite with probability one. Furthermore in many relevant cases, namely a large initial population, low death rate, or small transition rates leaving vertex 1, that the target is eventually populated is essentially equivalent to the vertex 1 population surviving. The probability that the vertex 1 population survives is obtained S1 Appendix (Eq. S7), and this quantity thus gives an approximation for the probability that the target population will ever arise, which is

$$\begin{array}{c} P\left(T<\infty \right)\approx 1-{\left(\beta /\alpha \right)}^{z}. \end{array}$$ (6)

This also explains the *β*/*α* term in (5), which arises due to lineages stemming from the initial vertex 1 cells going extinct. The corresponding approximate distribution for the target hitting time, when we condition that the vertex 1 population survives is given in S1 Appendix (Eq. S78). Differentiating the conditional hitting time distribution yields the density which is compared with simulations in Fig 3c.

Let us now discuss the time centring term *μ*, as given in Theorem 1. In the case of *β* = 0, *z* = 1, it can be easily seen (recall λ = *α* − *β*) that the median of our approximate distribution (5), is exactly *μ*. More generally, if we let *t*_1/2_ be the median time to hit the target, then if the vertex 1 population survives, we have

$$\begin{array}{c} t_{1/2}∼\mu -h\left(z\right). \end{array}$$ (7)

as the target seeding transition rates tend to 0. The shift *h*(*z*), whose precise form can be found in S1 Appendix (Corollary S2), has the behaviour

$$h(1)=0,\;h(z)∼\frac{1}{\lambda }\text{log}\frac{z\lambda }{\alpha },\;z\to \infty .$$ (8)

The shift exists as initiating the system with a larger number of cells at vertex 1, leads to the target being reached faster. In terms of notation *f* ∼ *g* means that *f*/*g* → 1, when the limit under consideration is taken.

We now return to the running example of the path graph setting which was introduced at the end of the General framework section. With *z* = 1, (7) yields that the median time for the target population to appear is

$$\begin{array}{c} t_{1/2}\approx \mu =\frac{1}{\lambda }\text{log}\frac{\lambda^{2}}{\alpha \nu (1,2)}+\frac{1}{\lambda }\sum_{i=2}^{N-1}\text{log}\frac{\lambda -\lambda (i)}{\nu (i,i+1)}, \end{array}$$ (9)

for small *ν*(*N* − 1, *N*). The first summand on the right hand side comes from waiting for the first transition from the vertex 1 population, while the second is due transitions between the remaining vertices. The first summand is distinct as the vertex 1 growth is the main cause of stochasticity, as discussed in the Materials and Methods section. The population growth and target hitting time is illustrated for the path graph case in Fig 3.

### Path distribution

We now move to our second question: which path leads to the target population arising? Our naive expectation is that paths with larger weights will be more likely. This simple conjecture turns out to be true. To show this we first introduce some notation.

To any existing cell, say at vertex *x*, we may define the cell’s vertex lineage. This tracks the vertices of cells in the ancestral lineage of the cell under consideration and is a sequence of the form (1, …, *x*). For example a cell with vertex lineage (1, 3, 4) is at vertex 4, and descended from a vertex 3 cell, who in turn descended from a vertex 1 cell. Any vertex lineage is a path in *G* and we denote the number of cells with vertex lineage *q* at time *t* as *X*(*q*, *t*). Note that while there may be multiple ancestors at the same vertex in a given cell’s ancestral lineage, for example several generations of vertex 3 cells before the first vertex 4 cell, we do not record these in the vertex lineage (more precisely *q*_*i*_ ≠ *q*_*i*+1_).

Now for any root to target path $p\in P_{1,N}$, that is a path of the form *p* = (1, *p*_2_, *p*_3_, …, *N*), the first time that *p* is traversed can be defined as

$$\begin{array}{c} T(p)=\text{inf}\{t\geq 0:X(p,t)>0\}. \end{array}$$

Observe that path *p* populating the target first is equivalent to *T*(*p*) = *T*. The question of which path initiated the target only makes sense if the target is eventually populated. To ensure that this occurs we condition on a surviving vertex 1 population, and hence let

$$\begin{array}{c} P_{1}\left(\cdot \right)=P\left(\cdot \;|\text{vertex}\;\text{1}\;\text{population}\;\text{survives}\right). \end{array}$$

Recall that the vertex 1 population surviving is used as a proxy for *T* being finite (the target is eventually populated), as discussed in the paragraph following (5). We can now state the answer to our second question, which is a special case of Theorem 2 presented below. The probability that the target is populated via path *p* is simply the path weight of *p*, suitably normalised, that is

$$\begin{array}{c} P_{1}\left(T\left(p\right)=T\right)\approx \frac{w(p)}{\sum_{q\in P_{1,N}}w\left(q\right)}=\frac{w(p)}{\phi_{N}} \end{array}$$ (10)

for small target seeding transition rates. One may ask not only if a particular path populated the target, but whether the target was initiated from a given set of paths, for an example see the Imperfect drug penetration: combination therapy section. In this case the probability that the target is populated via a particular set of paths is given by summing the normalised path weights over each path in the set.

If the vertex 1 population avoids extinction then *T*(*p*) will be finite for each root to target path *p*. Therefore instead of only asking which path populated the target first, one might be interested in whether a particular path is traversed significantly faster. For example, does multidrug resistance obtained via one of the paths in Fig 1a occur *t* days before resistance from the other path. In order to also consider this case, for a root to target path *p*, we define

$$\begin{array}{c} T\left(\neg p\right)=\text{min}\{T\left(q\right):q\in P_{1,N}\backslash \left\{p\right\}\}, \end{array}$$

that is the first time the target is reached along any path except *p*.

We can now quantify the probability of reaching the target via path *p* more than *t* time units before any other path. To avoid discussing a technical assumption, which excludes pathological scenarios, the theorem is stated in approximate form. The more precise version can be found in S1 Appendix (Theorem S3 in Section S5).

**Theorem 2**. *For small target seeding transition rates, and* t∈R,

$$\begin{array}{c} P_{1}\left(T\left(\neg p\right)-T\left(p\right)>t\right)\approx \frac{w(p)}{w\left(p\right)+e^{\lambda t}\sum_{q\in P_{1,N}\backslash \left\{p\right\}}w\left(q\right)}. \end{array}$$

Note that letting *t* = 0 gives (10). In the running example of a path graph we cannot ask which path seeded the target vertex. However we briefly illustrate the usefulness of the above theorem by considering the question of whether the target vertex is populated via a fitness valley (where by fitness valley we mean a path such that cells at the internal vertices have smaller growth rates than the root population).

**Fig 4:**
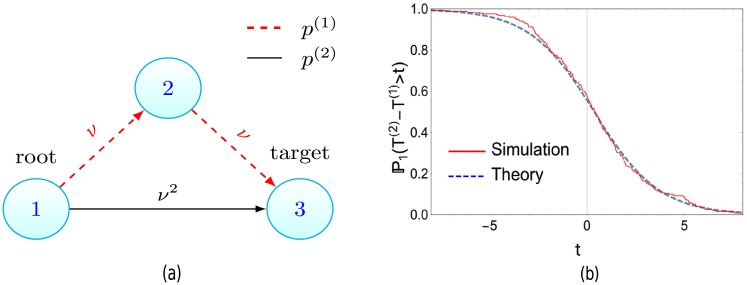
(a) Crossing valleys can be faster. Despite the red-dashed path containing a fitness valley (cells at vertex 2 have a reduced growth rate compared to the root population), the target population is more likely to arise via this path if λ − λ(2) < 1, as derived in (11). This is confirmed by the simulations displayed in (b), where λ − λ(2) = 0.8 < 1 and the Monte-Carlo estimate for $P_{1}\left(T^{\left(2\right)}>T^{\left(1\right)}\right)$ (the probability path 1 seeds the target first) is 0.58, while the analytic result yields 0.56. (b) Stochastic simulations of system in (a) vs theory (11). Parameters *α* = 0.9, *β* = 0.3, λ = 0.6, *α*(2) = 0.2, *β*(2) = 0.4, λ(2) = −0.2, *ν* = 0.1, runs = 250.

Let us consider the case depicted in Fig 4a where two paths exist to the target, a direct path and an indirect path. Here *N* = 3, with paths *p*^(1)^ = (1, 2, 3), *p*^(2)^ = (1, 3) and *ν* = *ν*(1, 2) = *ν*(2, 3), *ν*(1, 3) = *ν*^2^. Naively it may be expected that as the product of transition rates along both these paths are equal, and the indirect path (*p*^(1)^) contains a vertex with a fitness cost, the direct path (*p*^(2)^) is more likely. However, for small *ν*, Theorem 2 informs us that the target is populated via the indirect path *t* time units before the direct path, with probability

$$\begin{array}{c} P_{1}\left(T^{\left(2\right)}-T^{\left(1\right)}>t\right)\approx {\left[1+e^{\lambda t}\left(\lambda -\lambda \left(2\right)\right)\right]}^{-1} \end{array}$$ (11)

where *T*^(*i*)^ = *T*(*p*^(*i*)^). Thus the indirect path is more probable, that is $P_{1}\left(T^{\left(1\right)}<T^{\left(2\right)}\right)>P_{1}\left(T^{\left(2\right)}<T^{\left(1\right)}\right)$, if λ − 1 < λ(2).

Further applications of the results presented thus far will be given in the Applications section. Before this, we discuss the case when *G* is cyclic, and some extensions to the initial model for which our results still hold.

### Extensions

#### Cyclic graphs

In the above exposition we have assumed that *G* is acyclic, that is no path exists which contains cycles. We now briefly summarise how the results are altered if *G* is cyclic, with details given in S1 Appendix. We will however maintain that the target is a sink vertex (has no outgoing edges) and the root is a source vertex (has no incoming edges). The condition on the root is perhaps unnatural, as we now allow back transitions between vertices except to the root, but is required for technical reasons which are discussed in the S1 Appendix (see the remarks following the proofs of Theorem S1 and Proposition S2). We aim to drop this condition in future work.

First we introduce the *N* − 1 × *N* − 1 matrix *A* with entries

$$\begin{array}{c} a_{ij}=\{\begin{array}{c} \lambda (i)\;j=i\\ \nu (j,i)\;\text{if}\;(j,i)\in E\\ 0\;\text{otherwise} \end{array} \end{array}$$

and we denote the largest real eigenvalue of *A* as λ*.

In order that a modified version of Theorem 1 holds it is required that λ* = λ and λ* is a simple eigenvalue. This condition is guaranteed for acyclic graphs, and will also be satisfied if the transition rates throughout the graph are small enough (see Lemma S2 in the S1 Appendix). Assuming this eigenvalue condition, Theorem 1 holds but with *ϕ*_*N*_ replaced by summing particular elements of the λ-associated eigenvector of *A* (see Theorem S2 in S1 Appendix). Theorem 3, which concerns the population growth at the initial and intermediate vertices and is presented below in the Materials and Methods section, is similarly modified under the same condition (see Theorem S1 in S1 Appendix).

Turning to Theorem 2, we can now consider walks that contain cycles as opposed to only paths in the graph *G*. If we consider a particular set of root to target walks of a finite length, analogously to Theorem 2, we can ask whether the target is reached via one particular walk more than *t* units before any other walk in the specified set. A near identical result to Theorem 2 is obtained (see Proposition S2 in S1 Appendix). However the path weights should now be replaced by the walk weights (defined analogously to the path weight; the product of the transition rates along the walk divided by the fitness costs along the walk). In particular for two given walks of finite length we can still approximate the probability the target population arises via one of the walks versus the other.

**Fig 5:**
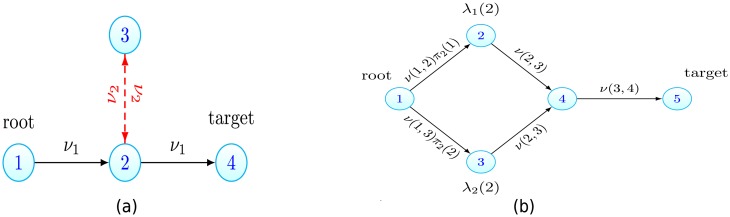
Example systems for which our extended results, given in the Extensions section, are useful. (a) Walks containing cycles can seed the target. The walk of length 4 that traverses the red edges with transition rate *ν*_2_ can still be faster than the length 2 path. See (12). (b) Incorporating a distribution of fitnesses. Take the path graph from Fig 2. Suppose that instead of a fixed λ(2), after the first transition cells have fitness λ_1_(2) or λ_2_(2) with probabilities *π*_2_(1), *π*_2_(2), with λ_*i*_(2) < λ. Then instead of the original path graph, we consider the enlarged graph shown here, where vertex 2 is replaced by two vertices with associated growth rates λ_1_(2), λ_2_(2). The transition rates to these new vertices are *ν*(1, 2)*π*_2_(*i*), where *ν*(1, 2) is the transition rate between vertices 1 and 2 in the original setting. All results can then be applied to the new enlarged graph.

For example we can use this to explore when a walk, containing a cycle, will seed the target before a path. Consider the graph and edge parameters displayed in Fig 5a. Let *p* = (1, 2, 4) and *ω* = (1, 2, 3, 2, 4). Despite the extra length, by using the modified Theorem 2, for small *ν*_1_ we see

$$\begin{array}{c} P_{1}\left(T\left(\omega \right)<T\left(p\right)\right)\approx (1+\frac{\left(\lambda -\lambda (2))(\lambda -\lambda (3)\right)}{\nu_{2}^{2}}{)}^{-1}. \end{array}$$ (12)

Hence the target can be seeded via the walk, if *ν*_2_ is large enough. On the other hand, by using the modified Theorem 2 it can be seen that when all the transition rates are small in the system, the target population will arise from a path and not a walk containing cycles (see Proposition S3 in S1 Appendix). This further justifies our focus on the acyclic case.

#### Distribution of fitness effects

Let us now expand on the comments in the General framework section where we claimed that having a finite distribution of fitness effects is covered by our model. This may be of interest, for example, when a cell acquires drug resistance via a mutation, but the cost of resistance is stochastic. More generally, suppose we wish to consider cells transitioning from vertex *x* to vertex *y* but require that the new growth rate of cells to be λ_*i*_(*y*) with probability *π*_*y*_(*i*), where *π*_*y*_(*i*) is a finite distribution and every λ_*i*_(*y*) < λ. Then we can simply replace the vertex *y* with new vertices associated to each of the λ_*i*_(*y*) and have cells transition from vertex *x* to the new vertices at rate *ν*(*x*, *y*)*π*_*y*_(*i*). We may freely apply the results of the Results section to the graph containing the new vertices and edges. See Fig 5b for an example.

#### Alternative offspring distribution

We have focused on the setting where cells can divide, die or transition, however Theorem 2 would also hold for a slightly more general branching process. Keeping the transition process as it is, we can have a vertex *x* cell being replaced by *j* vertex *x* cells at a rate *α*(*x*)*σ*_*x*,*j*_ where for each *x* between 1 and *N* − 1, (*σ*_*x*,*j*_)_*j*≥0_ is a probability distribution with finite second moment and mean ${\bar{\sigma }}_{x}$. We now have no need for *β*(*x*) as cell death is contained in *σ*_*x*,0_. This scenario may be relevant to the case of viral dynamics, where an infected cell can release multiple virions upon bursting [40]. In this setting if we redefine $\lambda \left(x\right)=\alpha \left(x\right)\left({\bar{\sigma }}_{x}-1\right)$ then the path distribution will be unchanged. This is due to the fact that the key result of [41] used in the proof of Theorem 3 holds then also. However Theorem 1 is no longer true in this setting. This is due to a lack of understanding of the large time behaviour of the root population (see the remarks following the proof of Theorem S1 in S1 Appendix).

## Discussion

Before discussing related work and summarising this study, we demonstrate how to apply the results of our Results section on some applications. The approximate formulas given in the Results section will be our key tools, and we now briefly remark on these.

The results of the Results section hold when the final transition rates in each path tends to 0. Therefore in our applications all transition rates, in particular the mutation and migration rates discussed below, will be taken to be small and statements are to be interpreted as approximations that hold true in this limiting regime. We also note that in our model, when transitions represent migrations, they also occur at cell divisions, (*x*) → (*x*), (*y*), instead of at any time, as (*x*) → (*y*). This might appear strange from a biological point of view, but the former formulation for transitions has been chosen, as it simplifies the mathematical treatment. More importantly, we expect that for small transition rates these formulations lead to very similar target hitting times. Indeed, simulations support this claim, as presented in Fig B of S1 Appendix. In all applications considered we shall neglect the role of back transitions. This is for simplicity and is in keeping with the previous works that we compare with. The effect of (finitely many) back transitions could be included by using our results on cyclic graphs.

### Applications

First we consider the impact of imperfect drug penetration on the emergence of resistance. Two recent publications have explored how resistance spreads in this setting, and have shown that poor penetration can accelerate resistance [30, 31]. We are able to recover and extend some of their findings in a rigorous fashion. Next, the ordering by which resistance-causing mutations accrue is investigated, firstly in the setting of cancer and then in bacterial infections. In the case of bacterial infections we examine how the risk of multidrug resistance depends on mutation rates, recovering simulation results in [26]. A particular aim of this section is to illustrate how to apply the results of our Results section, and so, for the readers’ convenience, we have collected the key formulas in Table 1.

**Table 1:** Key formulas needed for the Applications section. These approximations will be valid for small transition rates. The approximation for *t*_1/2_ assumes a large initial number of cells at the root, *z* ≫ 1. For small *z* see Corollary S2 in S1 Appendix.

Description	Formula	Reference
Weight of path *p*	$w\left(p\right)=\nu \left(p_{1},p_{2}\right)\prod_{i=2}^{l}\frac{\nu (p_{i},p_{i+1})}{\lambda -\lambda (p_{i})}$	Eq (2)
Total weight of target	$\phi_{N}=\sum_{p\in P_{1,N}}w\left(p\right)$	Eq (3)
Distribution of target hitting time	$P(T>t)\approx {\left(\frac{\lambda /\alpha }{1+e^{\lambda t}\phi_{N}\alpha /\lambda^{2}}+\beta /\alpha \right)}^{z}$	Eq (5)
Median of target hitting time	$t_{1/2}\approx \frac{1}{\lambda }\text{log}\frac{\lambda }{z\phi_{N}}$	Eq (7)
Probability target populated via path *p*	$P_{1}(T(p)=T)\approx \frac{w(p)}{\phi_{N}}$	Eq (10)

#### Imperfect drug penetration: Monotherapy

Our first two applications concern imperfect drug penetration and so the language introduced here will hold in the next section also. The first scenario we examine is exactly the same as that considered in [30].

We consider a pathogenic population, with cancer cells or bacteria as example populations, being treated with one or more drugs with imperfect penetration profiles. The imperfect penetration results in low drug concentration in spatial locations, such that cells in these locations may still proliferate. We term the cumulation of these spatial regions the *sanctuary*. Sanctuaries have been observed in both bacterial infections [42, 43] and tumours [44]. We consider resistance to have arisen when cells with sufficiently many mutations such that all therapies are ineffective come to exist in areas where the drugs have penetrated. The possibility of resistance occurring prior to treatment is excluded and we consider only the case when resistance arises from the sanctuary. Further, we do not consider cells acquiring mutations via gene transfer.

Throughout this section and the next we shall be comparing paths composed of mutation and migration events. Growing cells in the sanctuary will be the vertex 1 cells from the General framework section and thus have division and death rates *α*, *β*, with growth rate λ. The lack of exposure to drug motivates λ > 0. We shall assume all cells have division rate *α*. Across all cell types, i.e. regardless of location or drug presence, the per cell mutation and migration rates will be denoted *ν* and *m* respectively. We fix the initial number of cells in the sanctuary to be *z*. We will often suppose that *z* is dependent on how penetrative a particular drug is (as this will affect the sanctuary size).

In the case of monotherapy, there are two paths from which resistance might arise. Firstly, upon replication a cell in the sanctuary may produce a mutant able to grow in the presence of the drug. Then a mutated cell in the sanctuary can migrate to the drug compartment. We term this the mutation-migration path. Analogously we have the migration-mutation path which involves a susceptible cell migrating from the sanctuary, from whose progeny a resistant mutant emerges. We suppose a resistance-conferring mutation carries a fitness cost which will be denoted *s*. Resistance costs may not always exist but are well documented and often assumed in viruses, bacteria and cancer [33–35]. Due to the fitness cost of resistance the death rate of mutated cells in the sanctuary is increased to *β* + *s*, while the death rate of migrated susceptible cells in the drug compartment is *β* + *d*, with *s*, *d* > 0.

The first quantity to determine is the probability of escaping resistance, and its dependence on the initial number of cells in the sanctuary. The approximation of (6) shows that the probability resistance never occurs decreases exponentially with the initial number of cells in the sanctuary as $P\left(\text{resistance}\;\text{never}\;\text{occurs}\right)\approx {\left(\beta /\alpha \right)}^{z}$. For the rest of the discussion we will assume that resistance does occur. Further we suppose *z* ≫ 1, so that we may use the approximate form for *h*(*z*) given in (8). The case when *z* is small could be treated similarly using the exact expression for *h*(*z*).

We now ask when, and via which path, will resistant cells come to exist in the drug compartment? From the Results section we know the key quantities required to answer these questions are the path weights. For this system these are given by

$$\begin{array}{c} w\left(\text{mutation-migration}\;\text{path}\right)=\frac{\nu m}{s},\;w\left(\text{migration-mutation}\;\text{path}\right)=\frac{\nu m}{d}. \end{array}$$ (13)

If we now let *t*_1/2_ be the median time resistance occurs, then using the path weights with (7), we immediately have

$$\begin{array}{c} t_{1/2}\approx \frac{1}{\lambda }\text{log}\frac{\lambda^{2}}{\alpha }-\frac{1}{\lambda }\text{log}(\frac{\nu m}{s}+\frac{\nu m}{d})+\frac{1}{\lambda }\text{log}\frac{\alpha }{z\lambda } \end{array}$$ (14)

Of note is that the sanctuary size, mutation and migration rate all have equivalent impact. This simple result can now be explored under different biological scenarios.

For example, suppose we are currently using drug *A* with fitness cost *d*^(*A*)^ and penetration profile leaving *z*^(*A*)^ initial cells in the sanctuary. We consider instead using a second drug which results in sanctuary size *z*^(*B*)^ and has fitness cost *d*^(*B*)^. Let the median resistance times under the first and second drug be denoted $t_{1/2}^{\left(A\right)}$ and $t_{1/2}^{\left(B\right)}$ respectively. Then, using (14) and examining $t_{1/2}^{\left(B\right)}>t_{1/2}^{\left(A\right)}$, we find we should switch to drug *B*, if

$$\begin{array}{c} \frac{z^{\left(A\right)}}{z^{\left(B\right)}}>\frac{1+s/d^{\left(B\right)}}{1+s/d^{\left(A\right)}}. \end{array}$$ (15)

**Fig 6:**
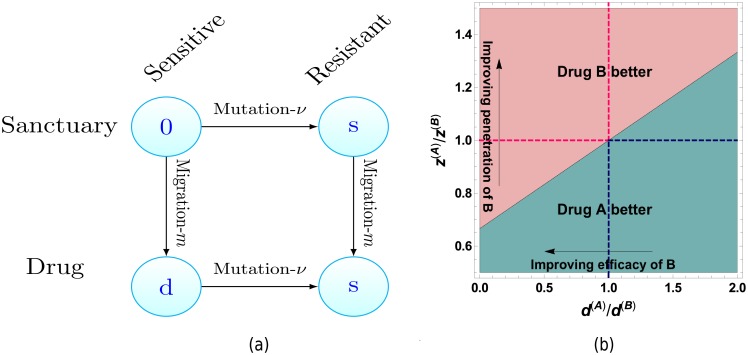
(a) In the case of monotherapy, there are two paths by which drug resistant cells can arise in the region containing the drug. Horizontal edges represent changes in genotype (gaining resistance) whereas vertical edges represent changes in spatial location. The labels of vertices are the fitness cost cells experience in that state and *μ*, *m* are the per cell mutation and migration rates. (b) Comparison of two drugs with differing efficacies and penetration profiles using (15). Arrows pertain to characteristics of drug *B* relative to drug *A*. Top left and bottom right quadrants illustrate region where drugs are trivially better, i.e. have superior penetration and efficacy. Parameters: *d*^(*A*)^ = 10^−2^, *s* = 5 × 10^−3^.

This inequality is illustrated in Fig 6b.

Note as *d*^(*B*)^ → ∞, implying drug *B* is far more effective, the condition tends to *z*^(*A*)^/*z*^(*B*)^ > (1 + *s*/*d*^(*A*)^)^−1^. This is due to the fact that when *d*^(*B*)^ is large resistance will always arise via the mutation-migration path (which we show below), so this provides an upper bound to the gain that can be obtained from increasing the drug efficacy. The above is under the assumption resistance will occur but note the probability of no resistance would decrease exponentially with larger sanctuary size.

Having discussed the time resistance will occur we now ask by which of the two paths it will occur. Let *T*^*νm*^ be the first time resistance arises via the mutation-migration path and *T*^*mν*^ be defined analogously for the migration-mutation path. Then Theorem 2 gives us

$$\begin{array}{c} P\left(T^{\nu m}<T^{m\nu }\right)\approx \frac{d}{s+d}. \end{array}$$

Hence resistance arising via the mutation-migration path is more probable, that is the above probability is greater than 1/2, if *d* > *s*. As most drugs are designed to at least halt cell growth, while many resistant cell lines can still proliferate, we expect this condition to be satisfied for most drugs. Note that neither *m* nor *ν* appear in the above expression as the numerator of both path weights is equal, see (13), and hence cancels.

A similar question was asked in [30]. There they considered an approximate stochastic process and sought the parameter regime such that *T*^*mν*^ stochastically dominated *T*^*νm*^ under the approximate process. For the full process considered here, we can ask the same question but on the limit of the centred (in the sense of Theorem 1) resistance times. The resulting condition for stochastic dominance is again *d* > *s*. The difference in the condition given here (*d* > *s*) and that stated in equation (28) in [30] exists as there *ν* is defined as the probability of mutation per division event. Accounting for this leads to the same condition.

#### Imperfect drug penetration: Combination therapy

There are at least two reasons for extending our analysis to include two drugs, say drugs *A* and *B*, being used in tandem. Firstly, combination therapies are widely implemented in HIV and bacterial infections, and there is a growing appreciation for their use in cancer [23, 45, 46]. Secondly, interesting new phenomena emerges, especially when a region still exists with only one drug present. The work presented in this section is inspired by the model and questions of [31].

**Fig 7:**
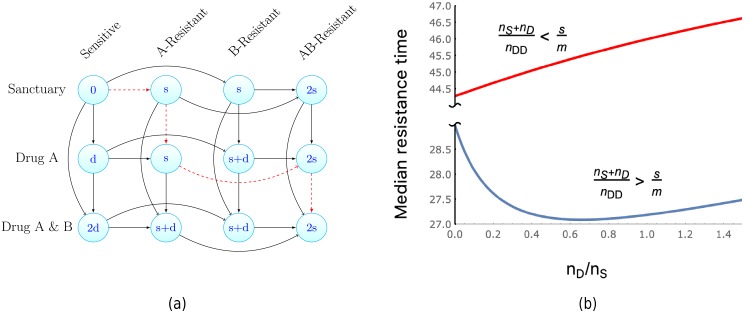
(a) Illustration of the paths to multidrug resistant cells arising in the region containing both drugs *A* and *B*, as in Fig 6. Note a spatial location exists containing only drug *A*. The red, dashed path is an example of stepwise evolution, where multidrug resistant cells come to exist in the sanctuary via the region containing only a single drug. The vertex labels display the fitness cost (increase to the cellular death rate) of cells in that state. (b) Exploration of the median time to resistance as the size of the drug *A* region increases relative to the sanctuary size. Here we plot *t*_1/2_, as given in Table 1, for the system displayed in (a). We observe that, for a subset of the parameter space, the existence of a single drug region can decrease the time to resistance, despite diminishing the sanctuary size. If both drugs penetrate the majority of the system and the drugs have a greater effect than the cost of resistance, this subset of parameter space is identified by the inequality (16). Within this parameter subset, resistance is fastest at a particular size of single drug compartment, given by (17). Parameters; (top) *ν* = 10^−6^, *m* = 0.05, *d* = 0.9, *s* = 10^−3^, λ = 0.4, *α* = 0.3, *n*_Tot_ = 107, *n*_DD_ = 0.9*n*_Tot_, γ = 10^−3^; (bottom) all same except *s* = 10^−2^.

We will only consider the case where the penetration profile for drug *B* is a subset of drug *A*, and thus a single drug region exists only for drug *A*. This allows us to clearly demonstrate the impact of unequal penetration profiles. In this setting there are 12 possible states that a cell may inhabit, defined by which of the drugs the cell is exposed to and the resistance profile it has obtained. These states are illustrated in Fig 7. We further assume that the mutation rate bringing resistance to either drug is *ν*. This is for simplicity only, and dealing with differing mutation rates is straightforward. Mutations that confer resistance will again have a fitness cost *s*, and sensitive cells coming into contact with drugs invokes a fitness cost *d*. These fitness costs increase the cellular death rate additively. The increase to the death rate for a cell in any given state is displayed as the vertex labels in Fig 7. Drug interactions, mutation specific fitness costs, and epistasis are neglected but may easily be included.

To investigate the effect of the single drug region, we introduce further notation. Let *n*_Tot_ be the number of cells that may reside in our system of interest (e.g. the entire patient or tumour) in the absence of drug. Further, let the regions where both drugs act, the single drug (drug *A*) acts and the sanctuary have their respective cell capacities denoted by *n*_DD_, *n*_D_, and *n*_S_. We assume that all these capacities are large, and hence we may still investigate the system with our branching process model. These capacities enter the dynamics of the process in the following manner. When a cell leaves the region it is in, at rate *m*, it now may migrate to one of two regions. Of these two regions, we specify that it migrates to a particular region at a frequency proportional to the regions capacity. For example a cell in the sanctuary migrates to the double drug region at rate *mn*_DD_/(*n*_D_ + *n*_DD_). Resistance mutations and the presence of the drug has the same additive effect as in the Imperfect drug penetration: monotherapy section.

Observe that there are 18 possible paths by which sensitive cells in the sanctuary can eventually produce fully drug resistant cells in the double drug compartment. To see this it is convenient to separate the paths into those that pass through the single drug compartment, which following [31] we term stepwise evolution, and those that involve migration from the sanctuary directly to the double drug compartment, which we collectively call direct evolution. For the direct evolution paths there are three transitions needed, mutations yielding resistance to drug *A* and *B* and then the migration to the double drug region. From the number of permutations of these events, this yields 6 direct evolution paths. In the stepwise evolution case four transitions are needed, both mutations and then the two requisite migration events. Notice that the migration from the sanctuary to the single drug region must precede the migration from the single drug region to the double drug region. This leads to 12 stepwise evolution paths. An exemplar stepwise evolution path is a mutation to drug *A*, followed by migration to the single drug region, then a mutation to drug *B*, and finally a migration to the double drug region. The edges of this path are red and dashed in Fig 7a.

Suppose, momentarily, that the penetration profiles of drugs *A* and *B* exactly match (but are still imperfect). In this case, resistance in the double drug region can be obtained only via direct evolution. If we now increase the penetration of drug *A* only, 12 new possible paths to resistance open up (the stepwise evolution paths of the previous paragraph), accelerating the time until resistance. However, the increased penetration also leads to a reduction in the sanctuary size, which should delay resistance. Hence a trade-off may exist with respect to the penetration profile of drug *A*. To study this we fix *n*_Tot_ = *n*_S_ + *n*_D_ + *n*_DD_ and specify that the initial number of cells in the sanctuary is related to its size via *z* = *γn*_S_ with 0 < *γ* ≪ 1. Further let *t*_1/2_(*n*_D_/*n*_S_) be the median time to resistance with fixed (*n*_D_/*n*_S_). Using the approximation provided by (7) we can explore the behaviour of *t*_1/2_(*n*_D_/*n*_S_). As can be seen in Fig 7b it is possible, in certain regimes of the parameter space, that the existence of the single drug compartment accelerates resistance (by reducing *t*_1/2_). We now seek the conditions under which this is the case. Brief derivations of the presented formulas below (16, 17, 18) are given in S1 Appendix (Section S8).

As mentioned previously, it is reasonable to expect the efficacy of either drug is greater than the cost to resistance. Also we may hope that both drugs penetrate the majority of the target system. This motivates taking *s* ≪ *d* and (*n*_S_ + *n*_D_) ≪ *n*_Tot_. Under this limit, by examining the sign of $\frac{d}{d(n_{D}/n_{S})}t_{1/2}\left(0\right)$, we find a simple condition for when increasing the single drug compartment accelerates resistance, namely

$$\begin{array}{c} \frac{n_{S}+n_{D}}{n_{\text{Tot}}}>\frac{s}{m}. \end{array}$$ (16)

However, when (16) holds, as *n*_D_/*n*_S_ increases, eventually a minimal resistance time is achieved. This represents a worse case scenario yielding fastest possible resistance. For *s* ≪ *d*, this minimal time occurs when

$$\begin{array}{c} \frac{n_{D}}{n_{S}}\approx 1-\frac{2sn_{\text{Tot}}}{m(n_{S}+n_{D})}. \end{array}$$ (17)

We now examine whether resistance arises via stepwise evolution or direct evolution. Let *T*^SE^ be the first time resistance occurs via stepwise evolution, and *T*^DE^ defined analogously for direct evolution. Then using Theorem 2, again with *s* ≪ *d*, we find

$$\begin{array}{c} P\left(T^{\text{SE}}<T^{\text{DE}}\right)\approx [1+\frac{s(n_{\text{DD}}+n_{S})}{mn_{D}}{]}^{-1}. \end{array}$$ (18)

Therefore the larger the proportional size of the single drug region, the more likely resistance will arise via stepwise evolution. A qualitatively similar result was derived in [31] (e-page 2878) by comparing the most likely stepwise evolution path with the most likely directed evolution path.

Furthermore, in [31] a minimal resistance time, in the sense of (17), was observed via simulations for the mutation_*A*_-migration-mutation_*B*_-migration path (the red and dashed path in Fig 7a). Using Theorem 2 we can show that this is the most probable (in the sense of the mode of the path distribution, see (10)) stepwise evolution path whenever *d* > *s*. Further, by differentiating the median time along this path, we see a minimal time occurs at *n*_D_ ≈ *n*_S_, which recovers the simulation result in [31] (see their Fig. 3). That this path is the most probable stepwise evolution path also gives insight into the recurring *s*/*m* term in (16) and (18). Relative to direct evolution this path has an extra migration step, so higher migration should increase the probability of seeding the target via this path. However fitness costs along this paths are incurred only via the cost of resistance, and a smaller *s* diminishes these fitness costs. While the above is motivated by and compared with the study [31], in contrast to that work, we do not consider a size-dependent branching process (the population was constrained by a large carrying capacity in [31]). Despite this we are able to derive similar qualitative, and indeed quantitative (e.g. the recovery of the simulation result discussed above), results. These features may be general properties of such models or are perhaps due to the size of the carrying capacity in [31].

#### Path to resistance in chronic myeloid leukemia

Let us discuss the example application of [25], concerning the mechanism by which resistance to the targeted therapy imatinib occurs in chronic myeloid leukemia (CML). The two main resistance mechanisms are via gene amplification of the BCR-ABL fusion gene, which drives the CML, or a point mutation resulting in a modification to the target protein of imatinib. Approximately 100 point mutations have been identified conferring resistance, and a rough estimate for the probability of each of these occurring during a cell division is 10^−7^ [47]. Thus the probability of a point mutation conferring resistance is approximately 10^−5^ per division. The analogous quantity for resistance-causing gene amplifications, in a similar system, was found to be approximately 10^−4^ [48]. With the division rate of leukemic stem cells as *α* = 0.008 day^−1^ [49] we have the rate of point mutations as *ν*_*pm*_ = 8 × 10^−8^ day^−1^, while the rate of resistance due to gene amplifications is *ν*_*ga*_ = 8 × 10^−7^ day^−1^. Therefore, using this information alone, the probability of resistance arising from a point mutation first is estimated to be 1/11. However, in the majority of cases, the primary mechanism of resistance is found to be point mutations [50].

Within our framework there are two possible explanations we can explore, both related to the reproductive capabilities of resistant cells. The first is simply that more lineages of amplified cells go extinct. The paragraph directly following (1) indicates how to treat this case. Indeed, if *ρ*_*pm*_ is the average survival probability for a population initiated by a single cell with a point mutation and *ρ*_*ga*_ the analogous quantity for gene amplification, then we find resistance arising first from a point mutation is more probable if *ρ*_*pm*_ > 10*ρ*_*ga*_. This is possible as point mutations may be deleterious or advantageous [47] whereas gene amplifications appear deleterious [51].

The second explanation, that suggested in [25], is that several gene amplifications are required to attain resistance. Suppose a gene amplification increases the death rate of cells by *s* per day. Then if two gene amplifications are required, ignoring the survival probability from before, the probability that resistance arises via point mutation is now

$$\begin{array}{c} \frac{\nu_{pm}}{\nu_{pm}+\nu_{ga}^{2}/s}={\left(1+8\times 10^{-6}/s\right)}^{-1}. \end{array}$$

Resistance via points mutations is now more likely if *s* > 10^−6^. Note that Fig 4a serves as a schematic for this scenario, reinterpreting the fitness valley path as resistance arising via gene amplifications and the direct path relating to resistance via point mutations. Further explanations for the primacy of point mutation mediated resistance exist and the assumptions above may not hold. But when they do hold, our results offer a clear framework to investigate such questions.

#### Antibacterial multidrug resistance

A timely problem is that posed by the emergence of multidrug resistant bacteria. We now explore some specific examples of our general framework that pertain to this issue. In particular we consider the accumulation of resistance-conferring mutations as a bacterial colony grows in the absence of drugs. As before we assume resistance incurs a fitness cost and thus the wild-type (unmutated cells) has the highest growth rate. Using Theorem 2 and measured fitness costs and mutation rates we can predict the most likely path to multidrug resistance.

We consider the acquisition of resistance to both rifampicin and streptomycin in *P. aeruginosa*. Recently the fitness costs associated with resistance to rifampicin [52] and streptomycin [53] have been reported. Fitness costs were determined by the maximum growth rate of the bacteria undergoing exponential growth on nutrient-rich plates. From [53] the ratio of streptomycin resistant bacteria to the wild-type’s growth rate was 0.71 (here the growth rate of the wild-type is estimated from Fig. 2.a in [53]). The same value for resistance to rifampicin, in [52], was 0.88. Let *T*^*RS*^ be the time when multidrug resistance emerges assuming rifampicin resistance is acquired first, and *T*^*SR*^ defined analogously for streptomycin resistance first. Then from Theorem 2, we have that

$$\begin{array}{c} P\left(T^{RS}<T^{SR}\right)\approx \frac{1-0.71}{\left(1-0.88)+(1-0.71\right)}=0.7. \end{array}$$

Hence, under this model, we would predict that rifampicin resistance emerges first en route to multidrug resistance. We note that above we have taken the average fitness costs associated with the differing antibiotics. Fluctuations have not been taken into account.

Instead of considering the the acquisition of resistance to different drugs within a bacterial strain, we can also examine the impact of differing mutation rates across strains. In [26] the heightened occurrence of multidrug resistance in lineage 2 vs lineage 4 from *M. tuberculosis* was investigated. Considering in particular resistance to rifampicin and isoniazid, via simulations they deduced that the heightened occurrence of resistance was due to elevated mutation rates in lineage 2 strains. The model simulated can be considered a discrete time counterpart to ours in the case of two paths of length 2 (see Fig 1a). One of the main quantities of interest was the relative probability of resistance, when the only difference between the strains was the mutation rates. For a particular strain, initiated with one wild-type cell with wild-type division and growth rates, *α*, λ, suppose we have mutation rates *ν*_R_, *ν*_I_ to rifampicin and isoniazid, each incurring the same fitness cost *s*. Then from Theorem 1, we have

$$\begin{array}{c} P\left(T\leq t\right)\approx 1-(\frac{\lambda /\alpha }{1+e^{\lambda (t-\mu )}}+\beta /\alpha ),\;\mu =\frac{1}{\lambda }\text{log}\frac{\lambda^{2}}{\alpha }+\frac{1}{\lambda }\text{log}\frac{s}{2\nu_{R}\nu_{I}}. \end{array}$$ (19)

For lineage-*i* (*i* = 2, 4) let $\nu_{R}^{\left(i\right)}$ and $\nu_{I}^{\left(i\right)}$ be the mutation rates to rifampicin and isoniazid. As in [26], keeping all other parameters equal between the lineages we have the relative probability of resistance

$$\begin{array}{c} \frac{P(T^{\left(2\right)}\leq t)}{P(T^{\left(4\right)}\leq t)}\approx \frac{\nu_{R}^{\left(2\right)}\nu_{I}^{\left(2\right)}}{\nu_{R}^{\left(4\right)}\nu_{I}^{\left(4\right)}}\frac{\left(1+2\nu_{R}^{\left(4\right)}\nu_{I}^{\left(4\right)}e^{\lambda t}\alpha /\lambda^{2}s\right)}{\left(1+2\nu_{R}^{\left(2\right)}\nu_{I}^{\left(2\right)}e^{\lambda t}\alpha /\lambda^{2}s\right)}. \end{array}$$

For times such that $\nu_{R}^{\left(i\right)}\nu_{I}^{\left(i\right)}e^{\lambda t}⪡1,\;i=2,4$, we see the relative probability is simply $\nu_{R}^{\left(2\right)}\nu_{I}^{\left(2\right)}/\nu_{R}^{\left(4\right)}\nu_{I}^{\left(4\right)}$. Taking the mutation rates measured in [26], this gives the relative probability of 22.06. This agrees with the simulation results in [26] who reported an approximately 22 fold increase. The same problem was treated in [38] but for multidrug resistance to two drugs in a fixed order (i.e. resistance to rifampicin followed by isoniazid). Staying in the regime $\nu_{R}^{\left(i\right)}\nu_{I}^{\left(i\right)}e^{\lambda t}⪡1$, if in (19), we replace the 2 by 1 (as only one path to resistance), and alter both λ^2^/*α* by λ, and *e*^λ*t*^ with *M*, our expression is the same to leading order as that given in [38] (equation 2) for the probability of resistance in a population of size *M*. This difference between fixed population size and fixed time results is the same as given in [16, 54].

### Concluding remarks

#### Related work

Many previous studies have considered the probability of particular cell type emerging in a growing population, typically by a fixed time after the process starts or when the total population reaches a given size. The majority deal with the target population being one or two transitions away from the initial population (the root vertex in this paper) [16, 18, 23, 38, 55–58]. In particular we single out the pioneering work of Luria and Delbrück [55], which demonstrated the spontaneous nature of mutations by combining an appropriate mathematical model with bacterial experiments on phage resistance. The original model of [55], and its various incarnations [59, 60] have been extensively studied [16, 57, 61–65]. Its fully stochastic formulation, which is identical to our model for two vertices, is due to Bartlett [60]. This full model admits an explicit solution [57], and the model’s asymptotic behavior has been recently explored [16, 64]. One of the simplest quantities of interest is the probability of no resistant cells at a fixed time, often called *p*_0_. It is a closely related quantity to our target hitting time described in (5), that is no resistant cell arises by a fixed time. Understanding *p*_0_ provides a method to infer the per cell-rate at which resistance-conferring mutations are acquired, often termed the *p*_0_-method [66].

Some notable exceptions which consider the target population being greater than two transitions away are [25, 31, 54, 67–70] (ref. [31] is discussed above in the Imperfect drug penetration: combination therapy section). In [25], the same model as that presented here is numerically explored when all vertices have the same fitness (*α*(*x*) = *α*, *β*(*x*) = *β* for all vertices *x*), and the implications on multidrug therapy failure is emphasised. An efficient numerical method to compute the distribution of target hitting time and path probabilities via the iteration of generating functions is given in [68] with a focus on cancer initiation. Both [54, 67] are motivated by the accumulation of driver mutations in cancer, and so each transition leads to a fitness increase. For [67] the mean time of the *k*th driver mutation derived and compared to genetic data. The distribution of the time until the *k*th driver is sought in [54], whose methods are the closest to those used in this paper. There the authors employed an appealing approximation of the model studied in this paper in the path graph case (the approximation is that the seeding rate into vertex *x* + 1 from vertex *x*, which is exactly *ν*(*x*, *x* + 1)*Z*_*x*_(*t*), is approximated by $\nu \left(x,x+1\right)W_{x}^{*}e^{\lambda (x)t}$, for some sensibly chosen random variables $W_{x}^{*}$). Notably, again for small transition rates, the functional form of the distribution of the target hitting time is the same as that given in Theorem 1, however with a different median. The altered median derived in [54] demonstrates that when transitions lead to a fitness increase, the growth rates associated with the intermediate vertex populations have a far greater effect on the target hitting time when compared to the setting considered in this paper. The same type of approximate model was further used in [69] to investigate the target hitting time when transitions bring a fitness increase which is a random variable. Target hitting times on a path graph for a branching process with general offspring distribution, were also discussed in the very recent paper [70], but their main explicit results exclude cell death, and hold when successive transition rates along the path are assumed to be getting (infinitely) stronger.

The model and questions considered in this paper also arise frequently in the evolutionary emergence or evolutionary escape literature [71–75], with the notable distinction that in the evolutionary escape setting the root and intermediate vertex populations are destined to go extinct (λ(*x*) < 0 for 1 ≤ *x* ≤ *N* − 1). This scenario is of interest when, for example, a homogeneous, pathogenic population (all residing at the root vertex in the language used in the present study) is treated with a therapy and must acquire sufficiently many mutations away from the root so as to become resistant (populate the target vertex). As in the setup of the present paper, the target hitting time is strongly controlled by the growth of the root population, *Z*_1_(*t*), which has positive growth rate, λ > 0, the target hitting times in the evolutionary escape setting are distinct from those given in the Time until target vertex is populated section. However for the escape probability (which is the probability of reaching the target), if there are multiple paths from the root to the target, the contribution of each path to the escape probability (termed the path value in [71, 72]) has an expression strikingly similar to the path weights discussed here for small transition rates (compare (2) with Eqs 6a-c in [72]). We might conjecture that for a specific path, the path value, as given in [72], is the unnormalised probability of reaching the target via the specified path, as we have demonstrated is the case with the path weights in Theorem 2 (this is implied in [72] Sec 2.5). Further connections surrounding the path distribution in these differing regimes is an interesting avenue for future work.

#### Summary

In this article we have considered a continuous time birth-death process with transitions. The time until a population of a particular target state arises was investigated. This target state is accessible via one or more transitions from the initial population. Which sequence of transitions leads to the target population arising was explored. This gave rise to a probability distribution over the paths composed of transitions between differing vertices. Motivated by applications, the setting when the initial population was most fit was focused on. The important factor in determining whether the target was reached via a specific path was seen to be the weight of the path. The weight of the path is composed of the transition rates and fitness costs associated with the intermediate vertices along the considered path.

Our primary contributions are the simple, explicit formulas for the target hitting time and the distribution of paths to the target, which are valid for small transition rates. When compared with previously given expressions for hitting times in branching processes, due to the reduction in complexity, we believe our results are easily interpretable and widely applicable. Further, to the best of our knowledge, the path distribution presented is the first analytic characterisation of evolutionary paths through a multitype branching process. The utility of our formulas was demonstrated on a variety of scenarios pertaining to the emergence of drug resistance. The biological relations revealed would have been difficult to deduce by a simulation based approach.

A shortcoming of our model is that the potential for unlimited growth and the lack of interactions between the cells is unrealistic. However in systems with large carrying capacities and abundant resources, our framework provides a convenient approximation and has been widely used. One possible route for assessing the importance of these neglected factors is to embed our questions in a similar setup to that used in the recent work [76], which included competition between cell types and a carrying capacity which the population could fluctuate around. The initial conditions in [76] were such that the model does not represent a growing population, so we do not compare their findings with the results of our Results section. Embedding our questions in a variant of the model of [76], such that the population is growing, might offer insight on the effect of these neglected aspects. Whenever these factors are deemed unimportant, or as a starting point for more complex models, this article provides quick and accessible results which may be used to guide and develop biological insight.

## Materials and methods

### Population growth

The main ingredient in proving Theorem 1, and consequently Theorem 2, is the long-time behaviour of the population of the initial and intermediate vertices. In this section we discuss this asymptotic behaviour.

Firstly we extend the definition of the total path weight given in the General framework section. Let the set of paths between the root and vertex *x* be denoted $P_{1,x}$. Then using the definition of path weight (2), we let the total weight for vertex *x* be $\phi_{x}=\sum_{p\in P_{1,x}}w\left(p\right)$ for 2 ≤ *x* ≤ *N*. Further, let us denote the ratio of the total weight to fitness cost for vertex *x* as

$$\begin{array}{c} \Phi_{x}=\;\frac{\phi_{x}}{\lambda -\lambda (x)} \end{array}$$

for 2 ≤ *x* ≤ *N* − 1. To allow us to conveniently describe the growth at vertex 1 also, we set Φ_1_ = 1.

The target population can only be founded by transitions from cells residing at the neighbouring vertices (vertices connected to the target by an edge). Therefore, understanding the population growth of cells at these vertices is needed to discuss the timing and manner in which cells at the target arise. At large times, this understanding is provided by the following theorem, concerning the population at the initial and intermediate vertices (it is useful to recall that *Z*_*x*_(*t*) is the number of cells at vertex *x* at time *t* and that we initiate with *z* cells at vertex 1). It follows from a more general result due to Janson [41], which we have tailored to our particular setting.

**Theorem 3**. *With probability one*

$$\begin{array}{c} \lim_{t\to \infty }e^{-\lambda t}{\left(Z_{x}\left(t\right)\right)}_{x=1}^{N-1}=W{\left(\Phi_{x}\right)}_{x=1}^{N-1}. \end{array}$$

*Here W is distributed as the sum of K independent exponential random variables with parameter* λ/*α*, *where K is binomial with z trials and success probability* λ/*α*.

A short proof, demonstrating how Theorem 3 can be deduced from [41] is given in Section S2 of the S1 Appendix (see Theorem S1). The distribution of *W* may be called a binomial-Erlang mixture, being an Erlang distribution with a binomially distributed shape parameter. For the running example of a path graph on *N* vertices, we see that $\Phi_{x}=\prod_{i=2}^{x}\frac{\nu (i-1,i)}{\lambda -\lambda (i)}$ for 1 ≤ *x* ≤ *N* − 1. This is demonstrated in Fig 3a. We note that in the case of *N* = 3, Theorem 3 was previously given in [16] (Theorem 3.2).

Using Theorem 3 and that the immigration rate into the target vertex from any target neighbouring vertex *x* is *Z*_*x*_(*t*)*ν*(*x*, *N*) we are able to prove the results of our Results section. Full proofs are provided in S1 Appendix.

## Supporting information

S1 Appendix Mathematical details and proofs. (PDF)
