# Supplementary material for: Competing evolutionary paths in growing populations with applications to multidrug resistance
Source: PLoS Comput Biol. 2019 Apr 15;15(4):e1006866. doi: 10.1371/journal.pcbi.1006866 (PMC6483269; doi:10.1371/journal.pcbi.1006866)
Supplement: S1 Appendix — (PDF) [file pcbi.1006866.s001.pdf]

Supplementary material for competing evolutionary paths in growing populations with applications to multidrug resistance

Michael D. Nicholson [1¤*], Tibor Antal [2],

1 School of Physics and Astronomy, University of Edinburgh, Edinburgh, Scotland
2 School of Mathematics, University of Edinburgh, Edinburgh, Scotland

¤Current Address: Department of Data Sciences, Dana-Farber Cancer Institute, Boston, MA, USA
* mdnicholson5@gmail.com

The outline of this supplement is as follows. First we recap the model, introducing notation that will be needed. Next we prove versions of Theorems 1 and 3 from the main text, which do not require G to be acyclic. After this we specialise to the acyclic case. Staying with the acyclic case we prove Theorem 2 from the main text. Finally, how the path distribution is altered when cycles are permitted is considered.

S1 Model recap

We recap our framework. Our model is a specific form of multitype branching process [1] in which each population will evolve according to a Markovian linear birth-death process with transitions. We will assume each population is comprised of cells. As a conceptual framework we associate the multitype branching process with a simple, finite, rooted, directed, graph $G = (V, E)$ containing N vertices ($N = |V|$). Labels of the vertices take values in $\{1, \dots, N\}$ and E is a subset of the set of ordered pairs $\{(i, j) : i, j \in V, i \neq j\}$. We shall often refer to vertex 1 as the root and vertex N as the target. Each vertex is reachable from the root and the target is reachable from any other vertex. We further assume the root is a source vertex and the target vertex is a sink. Letting the set of incoming neighbours for vertex i be $\mathcal{N}^-(i) = \{k \in V : (k, i) \in E\}$ and the set of outgoing neighbours $\mathcal{N}^+(i) = \{k \in V : (i, k) \in E\}$, the previous assumption is $\mathcal{N}^-(1) = \mathcal{N}^+(N) = \emptyset$. Each type in the branching process is uniquely mapped to a vertex, and so the number of types is N. Hence any cell may be described by its type or the vertex associated to that type.

Take any cell at vertex x. We assume this cell divides (replaced by two identical copies) at rate $\alpha(x)$, dies (removed from system) at rate $\beta(x)$ and transitions to a cell at vertex y (replaced by an identical copy and a cell at vertex y) at rate $\nu(x, y)$ if edge (x, y) is contained in E. The growth rate of cells at vertex x will be denoted $\lambda(x) = \alpha(x) - \beta(x)$. The parameters associated with the vertex 1 population feature prominently and so for convenience we let $\alpha = \alpha(1)$, $\beta = \beta(1)$ and $\lambda = \lambda(1)$. All cells are independent. We will focus on the setting where the vertex 1 population is the most fit and has positive growth rate. Therefore, we henceforth assume that $\lambda > 0$ and for $2 \leq x \leq N - 1$, $\lambda(x) < \lambda$. We do not specify the relative fitness of the vertex N population. The cell level dynamics may summarised as

$$(x) \to \begin{cases} (x), (x) & \text{rate } \alpha(x) \\ \varnothing & \text{rate } \beta(x) \\ (x), (y) & \text{rate } \nu(x, y) \text{ if } (x, y) \in E \end{cases} \tag{S1}$$

where (x) represents a cell at vertex x and $\varnothing$ symbolises a dead cell. At a population level, the number of cells at vertex x at time t will be denoted $Z_x(t)$. We shall always assume $Z_x(0) = z\delta_{x,1}$, where $\delta_{x,y}$ is the Kronecker delta function. The population growth of the initial and intermediate vertices is crucial and so the notation $\mathcal{Z}(t) = (Z_x(t))_{1 \leq x \leq N-1}$ will be used.

We also recap some relevant definitions from the main text that will be used repeatedly. First, the target hitting time is defined to be

$$T = \inf\{t \geq 0 : \ Z_N(t) > 0\}. \tag{S2}$$

Now let the set of paths between the root and any vertex $x \in V$ be be denoted $\mathcal{P}_{1,x}$. Then we define the weight of the path $p \in \mathcal{P}_{1,x}$ to be

$$w(p) = \nu(p_1, p_2) \prod_{i=2}^{l} \frac{\nu(p_i, p_{i+1})}{\lambda - \lambda(p_i)}, \tag{S3}$$

Throughout the empty product is set to 1. Further, we let the total weight of the the vertex x be

$$\phi_x = \sum_{p \in \mathcal{P}_{1,x}} w(p). \tag{S4}$$

S2 Population growth

In this section we prove Theorem 3 from the main text, but G is not assumed to be acyclic. Specialising to the acyclic case is in Section S4.

As the root vertex is a source, and hence no type transitions into vertex 1 cells, $Z_1(t)$ follows a linear birth-death process. Its asymptotic behaviour is described by the following classic result [1,3].

Lemma S1.

$$\lim_{t \to \infty} Z_1(t) e^{-\lambda t} = W \quad a.s. \tag{S5}$$

Here W is distributed as the sum of K independent exponential random variables with parameter λ/α, where K is binomial with z trials and success probability λ/α.

W, as given in the above theorem, is positive if and only if the vertex 1 population survives (almost surely). It will be convenient to define this event and so we let

$$S_1 = \{Z_1(t) > 0 \text{ for all } t \geq 0\}. \tag{S6}$$

From the distribution of W, in particular using that $\mathbb{P}(S_1) = \mathbb{P}(W > 0) = \mathbb{P}(K > 0)$ with K as in Lemma S1, we immediately have

$$\mathbb{P}(S_1) = 1 - (\beta/\alpha)^z. \tag{S7}$$

To obtain a similar result to Lemma S1 for $\mathcal{Z}(t)$, we recall the $N-1 \times N-1$ matrix A from the Extension section of the main text, with entries

$$a_{ij} = \begin{cases} \lambda(i) & j = i \\ \nu(j,i) & \text{if } (j,i) \in E \\ 0 & \text{otherwise.} \end{cases} \tag{S8}$$

Note that the off diagonal elements of the transpose of A are positive whenever the corresponding element of the adjacency matrix of G is one.

We will be using a result from [5] which holds when the largest real eigenvalue of A, λ^*, is simple (i.e. has algebraic multiplicity 1) and equal to λ. Below we give two sufficient conditions for this to be true. The second is motivated by applications when typically all transition rates are small.

Lemma S2. *Two sufficient conditions such that $\lambda^* = \lambda$ and is simple are: 1) G is acyclic. 2) For all $2 \leq i \leq N-1$, $\lambda(i) + \sum_{k \in \mathcal{N}^-(i)} \nu(k,i) < \lambda$.*

Proof. 1) As G is directed and acyclic, a relabelling of the vertices exists (often called the topological sorting, see [2] chapter 22.4) such that the underlying adjacency matrix of the relabelled graph is upper triangular. We use this relabelling with vertex 1 as first in the labelling, π. Thus π is a permutation of $\{1, \ldots, N\}$ with $\pi(1) = 1$. The permutation matrix associated with π and its transpose are

$$P_1 = \begin{bmatrix} \mathbf{e}_{\pi(1)}^{\mathrm{T}} \\ \vdots \\ \mathbf{e}_{\pi(n)}^{\mathrm{T}} \end{bmatrix}, \quad P_1^{\mathrm{T}} = \left[\mathbf{e}_{\pi(1)}, \cdots, \mathbf{e}_{\pi(N)}\right]. \tag{S9}$$

Under π we have the new matrix

$$A' = P_1 A P_1^{-1}. \tag{S10}$$

Due to the relation between the adjaceny matrix of G and A, we see A' is lower triangular and has the $\lambda(i)$ as diagonal elements. Thus λ is the largest eigenvalue of A'. As A and A' are similar matrices, and hence share eigenvalues, we can conclude the statement.

2) By considering the left eigenvector $\mathbf{e}_1^{\mathrm{T}}$, λ is indeed an eigenvalue. We now demonstrate all other eigenvalues have real part smaller than λ, which implies λ is simple. Observe that the assumed condition implies the bound on the row sums

$$\sum_{j=1}^{N-1} a_{i,j} < \lambda. \tag{S11}$$

As A is reducible (due to the root being a source vertex) there exists a permutation matrix P_2 such that

$$P_2 A P_2^{-1} = \begin{pmatrix} \lambda & & & \\ R_{2,1} & R_{2,2} & & 0 \\ \vdots & & \ddots & \\ R_{M,1} & \ldots & & R_{M,M} \end{pmatrix} \tag{S12}$$

with each submatrix $R_{i,i}$ square and irreducible and $2 \leq M \leq N-1$ (see [7] section 2.3). The eigenvalues of A comprise λ and the eigenvalues of the $R_{i,i}$. For any of the $R_{i,i}$ let $r_j^{(i)}$ be sum of the jth row of $R_{i,i}$. By Gershgorin's Disc Theorem ([7] Theorem 1.5) the real part of the eigenvalues of $R_{i,i}$ are bounded by $\max_j\{r_j^{(i)}\}$. Further, the bound (S11) is preserved under permutations and so each $r_j^{(i)} < \lambda$. As this holds for each $R_{i,i}$ the claim is shown. □

Henceforth we assume either of the conditions given in Lemma S2 hold and thus $\lambda^* = \lambda$ and is simple. Due to this assumption, results of [5] show that the vertex 1 cells drives the entire population growth. To state this result, we introduce the vector ψ with

$$\psi_i = \alpha(i) + \beta(i) + \sum_{k \in \mathcal{N}^+(i)} \nu(i,k). \tag{S13}$$

Now let $\tilde{u}, \tilde{v}$ be the left and right eigenvectors of A corresponding to λ, scaled such that

$$\psi \cdot \tilde{v} = 1, \quad \tilde{u} \cdot \tilde{v} = 1. \tag{S14}$$

Note, from the structure of A, $\tilde{u}_i > 0$ if $i = 1$ and 0 otherwise. Further let

$$\Phi = \tilde{u}_1 \tilde{v} \tag{S15}$$

Then the following result, which is the more general form of Theorem 3 in the main text, holds.

Theorem S1. *With W as in Lemma S1,*

$$\lim_{t\to\infty} e^{-\lambda t}\mathcal{Z}(t) = W(\Phi_x)_{x=1}^{N-1} \quad a.s. \tag{S16}$$

Proof. We demonstrate how to apply the result in [5] to our setting. From Theorem 3.1 in [5], with probability one

$$\lim_{t\to\infty} e^{-\lambda t}\mathcal{Z}(t) = \hat{W}\tilde{v} \tag{S17}$$

where $\hat{W}$ is a non-negative random variable, with as yet unknown distribution. However from Lemma S1 almost surely $e^{-\lambda t}Z_1(t) \to W$, with the distribution of W given in the lemma. This implies $\hat{W} = W/\tilde{v}_1 = \tilde{u}_1 W$ with the last equality following from (S14) and that only the first entry of $\tilde{u}$ is positive. □

The proof of Theorem S1 indicates our primary reason for taking the root vertex to be a source (no incoming edges). If transitions were permitted into the vertex 1 population, but still $\lambda^* = \lambda$ and is simple, then (S17) still applies but we do not know the distribution of $\hat{W}$. Additionally, regardless of whether the root is a source, if we consider the the more general offspring distribution discussed in the Extensions section of the main text, we similarly would have no relevant knowledge of the distribution of $\hat{W}$ (the Laplace transform of $\hat{W}$ is known to satisfy an implicit integral equation [1], but we cannot see how to use this information here).

S3 Time until target vertex is populated: general case

We now turn our attention to T, the time at which the target population arises, as defined in (S2). So that we can control all target seeding transition rates we let

$$\nu^* = \max_{i\in\mathcal{N}^-(N)} \nu(i,N) \tag{S18}$$

i.e. the largest of the transition rates into the target. We further give the definition of μ in this more general setting as

$$\mu = \frac{1}{\lambda}\log\frac{\lambda^2}{\alpha\sum_{i\in\mathcal{N}^-(N)}\nu(i,N)\Phi_i}. \tag{S19}$$

Then the following holds.

Theorem S2. *Let W be as in Theorem S1, then with probability one*

$$\lim_{\nu^*\to 0}\mathbb{P}(\lambda(T-\mu) > t|(\mathcal{Z}(t))_{t\geq 0}) = \exp\left(-e^t W\lambda/\alpha\right). \tag{S20}$$

It follows that

$$\lim_{\nu^*\to 0}\mathbb{P}(\lambda(T-\mu) > t) = \left(\frac{\lambda/\alpha}{1+e^t} + \beta/\alpha\right)^z \tag{S21}$$

Proof. Let

$$\sigma = \sum_{i\in\mathcal{N}^-(N)} \nu(i,N)\Phi_i. \tag{S22}$$

Then, as for given $(\mathcal{Z}(t))_{t\geq 0}$ immigration into vertex N occurs as a non-homogeneous Poisson process,

$$\mathbb{P}(T-\lambda^{-1}\log(\sigma^{-1}) > t|(\mathcal{Z}(t))_{t\geq 0}) = \exp\left(-\int_0^{t+\lambda^{-1}\log(\sigma^{-1})}\sum_{i\in\mathcal{N}^-(N)}\nu(i,N)Z_i(s)\,ds\right). \tag{S23}$$

The variable change $u = s - \lambda^{-1}\log(\sigma^{-1})$ yields

$$\exp\left(-\int_{-\lambda^{-1}\log(\sigma^{-1})}^{t} \sum_{i\in\mathcal{N}^-(N)} \nu(i,N) Z_i(u+\lambda^{-1}\log(\sigma^{-1}))\,du\right). \tag{S24}$$

Now observe that for any $i \in \mathcal{N}^-(N)$, by multiplying by $1 = \frac{e^{\lambda(u+\lambda^{-1}\log(\sigma^{-1}))}}{e^{\lambda(u+\lambda^{-1}\log(\sigma^{-1}))}}$,

$$\nu(i,N)Z_i(u+\lambda^{-1}\log(\sigma^{-1})) = \frac{Z_i(u+\lambda^{-1}\log(\sigma^{-1}))}{e^{\lambda(u+\lambda^{-1}\log(\sigma^{-1}))}}\frac{\nu(i,N)e^{\lambda u}}{\sigma}. \tag{S25}$$

Consider the limit of (S25) as $\nu^* \to 0$, which results in $\log(\sigma^{-1}) \to \infty$. As the $(Z_i(t))_{i\in\mathcal{N}^-(N)}$ are independent of $(\nu(i,N))_{i\in\mathcal{N}^-(N)}$, Theorem S1 informs us that, almost surely,

$$\lim_{\nu^*\to 0} \frac{Z_i(u+\lambda^{-1}\log(\sigma^{-1}))}{e^{\lambda(u+\lambda^{-1}\log(\sigma^{-1}))}} = We^{\lambda u}\Phi_i \tag{S26}$$

Upon considering $(\sigma/\nu(i,N))^{-1}$, we see $\nu(i,N)/\sigma < \infty$ for each i. Hence taking limits across (S25) yields, with probability one,

$$\lim_{\nu^*\to 0} \sum_{i\in\mathcal{N}^-(N)} \frac{Z_i(u+\lambda^{-1}\log(\sigma^{-1}))}{e^{\lambda(u+\lambda^{-1}\log(\sigma^{-1}))}}\frac{\nu(i,N)e^{\lambda u}}{\sigma} = We^{\lambda u}\lim_{\nu^*\to 0}\sum_{i\in\mathcal{N}^-(N)}\frac{\nu(i,N)\Phi_i}{\sigma}. \tag{S27}$$

By the definition of σ this last sum is one and hence

$$\lim_{\nu^*\to 0} \sum_{i\in\mathcal{N}^-(N)} \nu(i,N)Z_i(u+\lambda^{-1}\log(\sigma^{-1})) = We^{\lambda u} \quad a.s. \tag{S28}$$

We seek to apply the dominated convergence theorem to the integral in (S24). The limit demonstrated in (S28) implies that for any realisation, we may find x such that for $\nu^* \le x$

$$\sum_{i\in\mathcal{N}^-(N)} \nu(i,N)Z_i(u+\lambda^{-1}\log(\sigma^{-1})) \le 2We^{\lambda u} \tag{S29}$$

which is integrable over $(-\infty, t]$. If $\nu^* > x$ then $\sigma > 0$. Thus the interval $[-\lambda^{-1}\log(\sigma^{-1}), t]$ is finite. As each $Z_i(t)$ is cadlag, it is bounded over finite intervals. Hence for all (ν^*, u) the integrand is dominated by an integrable function. Therefore, taking the limit of (S24) and using (S28) leads to, with probability one,

$$\lim_{\nu^*\to 0} \mathbb{P}(T - \lambda^{-1}\log(\sigma^{-1}) > t | (\mathcal{Z}(t))_{t\ge 0}) = \exp\left(-We^{\lambda t}/\lambda\right). \tag{S30}$$

Using that

$$\mu = \lambda^{-1}\log(\lambda^2/\alpha) + \lambda^{-1}\log(\sigma^{-1}) \tag{S31}$$

so that we centre appropriately, leads to the first stated result.

For the second statement, we must derive an expression for

$$\mathbb{E}\left[\exp\left(-e^t W\lambda/\alpha\right)\right] \tag{S32}$$

From Lemma S1

$$W\lambda/\alpha \overset{d}{=} \sum_{i=1}^{z} \chi_i \xi_i' \tag{S33}$$

where $\chi_i \sim \text{Bernoulli}(\lambda/\alpha)$, $\xi_i' \sim \text{Exponential}(1)$ and are independent. Thus

$$\mathbb{E}\left[\exp\left(-e^t W\lambda/\alpha\right)\right] = \left(\mathbb{E}\left[\exp\left(-e^t \chi_1 \xi_1'\right)\right]\right)^z. \tag{S34}$$

Using a one-step conditioning argument on χ_1 and considering the Laplace transform for a unit rate exponential variable leads to the stated result. □

Before proceeding, we comment on a limitation of our proof approach. A key point in the proof of Theorem S2 is that the stochastic process $(Z_i(t))_{1\leq i\leq N-1, t\geq 0}$ is independent of the target seeding transition rates $(\nu(i,N))_{i\in\mathcal{N}^-(N)}$. This permits the limit displayed in (S26) to hold. If we considered a limit where transition rates associated with non-target adjacent edges also tended to 0, the limit (S26) would not hold. This is as Theorem S1 is true for fixed $(\alpha(x))_{x=1}^{N-1}$, $(\beta(x))_{x=1}^{N-1}$ and transition rates associated with non-target adjacent edges (the parameters controlling the growth of the populations at vertices $1,\dots,N-1$). Our inability to alter these parameters is also the reason, that in a precise sense, our results do not cover the formulation of transitions where type x divide at rate $\alpha'(x)$, and then with probability $\nu'(x,y)$ a transition occurs to vertex y. In that formulation, taking the limit when $\nu'(x,N)$ tends to 0, for $x\in\mathcal{N}^-(N)$, alters the division rate of cells at vertices in $\mathcal{N}^-(N)$, again forbidding the use of Theorem S1. We hope to improve upon these limitations in future work.

The first statement in Theorem (S2) will be key to understanding the path distribution, discussed in the Path distribution section of the main text, which justifies its prominence. As it implies that, with probability one,

$$\lim_{\nu^*\to 0}\mathbb{P}(\exp\left[\lambda(T-\mu)\right]>t|(\mathcal{Z}(t))_{t\geq 0})=\exp\left(-tW\lambda/\alpha\right), \tag{S35}$$

the first statement in Theorem (S2) can be interpreted as indicating that if we consider the conditional distribution of T around μ, then rescale time, an exponential distribution appears.

As discussed in the Time until target vertex is populated section of the main text, it is natural to consider the distribution of T conditioned that it is finite (we cannot discuss which path populated the target vertex if $T=\infty$). However it is technically more convenient to condition on S_1, defined in (S6). The following proposition states that in many relevant cases, namely large initial population, low death rate or small transition rates leaving vertex 1. these events are similar. Below, the superscript c denotes the complement of the set.

Proposition S1.

$$\mathbb{P}(\{T<\infty\}^c\cap S_1)=0. \tag{S36}$$

$$\mathbb{P}(\{T<\infty\}\cap S_1^c)\leq\begin{cases}O((\beta/\alpha)^z), & \beta\to 0\\ O((\beta/\alpha)^z), & z\to\infty\\ O(\nu_1), & \nu_1\to 0\end{cases} \tag{S37}$$

where $\nu_1=\sum_{x\in\mathcal{N}^+(1)}\nu(1,x)$.

Proof. As $\mathbb{P}(\{T<\infty\}^c\cap S_1)=\mathbb{P}(T=\infty|S_1)\mathbb{P}(S_1)$ and $\mathbb{P}(S_1)>0$ we initially demonstrate

$$\mathbb{P}(T<\infty|S_1)=1. \tag{S38}$$

Firstly recall that with W as in Lemma S1, up to null events, $\{W>0\}=S_1$. Now T is the first arrival in a Cox process with intensity $\sum_{x\in\mathcal{N}^-(N)}\nu(x,N)Z_x(s)$, and hence $T<\infty$ with probability 1 on S_1 if

$$\int_0^\infty\sum_{x\in\mathcal{N}^-(N)}\nu_{x,N}Z_x(s)ds=\infty \tag{S39}$$

with probability 1 on S_1. Observe that (S39) is true as, by Theorem S1, for any realisation we may find $t_1\geq 0$ such that

$$\int_{t_1}^\infty\sum_{x\in\mathcal{N}^-(N)}\nu(x,N)Z_x(s)ds\geq 2^{-1}\int_{t_1}^\infty\sum_{x\in\mathcal{N}^-(N)}\nu(x,N)W\Phi_x e^{\lambda s}ds. \tag{S40}$$

As each $\Phi_x > 0$, due to each entry of $\tilde{v}$ also being positive [5], the right hand side of the above inequality is infinite a.s. on S_1 which gives (S38). It remains to bound $\mathbb{P}(\{T < \infty\} \cap S_1^c)$.

Let us introduce $Z^*(t) = \sum_{i \in \mathcal{N}^+(1)} Z_i(t)$ and

$$\tau = \inf\{t \geq 0 : Z^*(t) > 0\}. \tag{S41}$$

Note that we cannot hope to populate the target vertex without $Z^*(t)$ becoming positive. Therefore, $\{T < \infty\} \subseteq \{\tau < \infty\}$ which implies

$$\mathbb{P}(\{T < \infty\} \cap S_1^c) \leq \mathbb{P}(\{\tau < \infty\} \cap S_1^c). \tag{S42}$$

Hence our interest turns to the right hand side of the preceding inequality. The distribution of τ is invariant to the growth at vertices $i \in \mathcal{N}^+(1)$, and so we let $\alpha(i) = \beta(i) = 0$ for such i. The process $(Z_1(t), Z^*(t))$ is a two-type branching process where cells of the second type (that contribute to $Z^*(t)$) simply accumulate. As

$$\mathbb{P}(\{\tau < \infty\} \cap S_1^c) = (1 - \mathbb{P}(\{\tau = \infty\}|S_1^c))\mathbb{P}(S_1^c) \tag{S43}$$

$$= \mathbb{P}(S_1^c) - \lim_{t\to\infty} \mathbb{P}(Z_1(t) = 0, Z^*(t) = 0), \tag{S44}$$

and from (S7) we know $\mathbb{P}(S_1^c) = \beta/\alpha$, we now solve for the joint distribution of $(Z_1(t), Z^*(t))$.

For now assume $z = 1$. We introduce the generating functions

$$G_1(x, y, t) = \mathbb{E}[x^{Z_1(t)} y^{Z^*(t)} | (Z_1(0), Z^*(0)) = (1, 0)] \tag{S45}$$

$$G^*(x, y, t) = \mathbb{E}[x^{Z_1(t)} y^{Z^*(t)} | (Z_1(0), Z^*(0)) = (0, 1)]. \tag{S46}$$

Immediately $G^*(x, y, t) = y$ for all t. The backward Kolmogorov equation for G_1 is

$$\frac{\partial}{\partial t} G_1 = \alpha G_1^2 + \beta + \nu_1 G_1 G^* - (\alpha + \beta + \nu_1) G_1. \tag{S47}$$

By solving this equation (separation of variables with a partial fraction decomposition), and using the initial condition we find

$$\mathbb{P}(Z_1(t) = 0, Z^*(t) = 0) = G_1(0, 0, t) = \frac{r_2(1 - e^{\alpha(r_2 - r_1)t})}{1 - r_2 e^{\alpha(r_2 - r_1)t}/r_1}. \tag{S48}$$

With

$$r_1 = \frac{1}{2}\left(1 + q_1 + \nu_1' + \sqrt{(1 + q_1 + \nu_1')^2 - 4q_1}\right) \tag{S49}$$

$$r_2 = \frac{1}{2}\left(1 + q_1 + \nu_1' - \sqrt{(1 + q_1 + \nu_1')^2 - 4q_1}\right) \tag{S50}$$

where $q_1 = \beta/\alpha$ and $\nu_1' = \nu_1/\alpha$. Observe that $(1 + q_1 + \nu_1')^2 - 4q_1 > (1 - q_1)^2$ which implies $r_2 - r_1 < 0$. Hence

$$\lim_{t\to\infty} \mathbb{P}(Z_1(t) = 0, Z^*(t) = 0) = r_2. \tag{S51}$$

We now have expressions for the final terms in (S43) for $z = 1$. For $z \geq 1$, we can use independence, and this leads to

$$\mathbb{P}(\{\tau < \infty\} \cap S_1^c) = q_1^z - r_2^z. \tag{S52}$$

By examining the leading order with respect to the relevant variables we can conclude.

□

We remark that if we instead wish to look at the case when the vertex N population arises and does not go extinct then $\mathbb{P}(\text{All populations go extinct}) = \mathbb{P}(\tilde{T} = \infty)$ where $\tilde{T}$ is the first time the target is founded by a cell whose progeny does not go extinct. Recall that our results for T hold identically for $\tilde{T}$ so long as the mapping $\nu(x, N) \mapsto \nu(x, N)\lambda(x)/\alpha(x)$ for all $x \in \mathcal{N}^-(N)$ is applied . The probability of population extinction is known to be given by an implicit equation involving the offspring distribution's generating function [1]. This was the approach taken by [4]. The approximate solution for small transition rates was derived and agrees with the result presented above (see Eq. A.4a in Appendix A of [4]).

The above proposition yields $\mathbb{P}(T < \infty) \approx \mathbb{P}(S_1)$. Using this with (S7) gives Eq. 6 in the main text. We now give consider the distribution of T given S_1 (which we use as proxy for conditioning on a finite hitting time).

Corollary S1. *With the notation of Theorem S2,*

$$\lim_{\nu^* \to 0} \mathbb{P}(\lambda(T - \mu) > t | S_1) = \frac{\left[\lambda/\alpha(1 + e^t)^{-1} + \beta/\alpha\right]^z - (\beta/\alpha)^z}{1 - (\beta/\alpha)^z} \tag{S53}$$

Proof. Starting from the first statement in Theorem S2, we only need apply $\mathbb{E}[\cdot|S_1]$. The distribution of $W\lambda/\alpha$ can be rewritten as

$$W\lambda/\alpha \overset{d}{=} \sum_{i=1}^{K} \xi_i' \tag{S54}$$

where ξ_i' are rate one exponential variables, and $K \sim \text{Binomial}(z, \lambda/\alpha)$, independent of all ξ_i'. Conditioning on S_1 ensures that $K > 0$.

Thus we must calculate

$$\mathbb{E}\left[\exp\left(-e^t W\lambda/\alpha\right)|S_1\right] = \mathbb{E}\left[(1 + e^t)^{-K} | K > 0\right]. \tag{S55}$$

The binomial theorem leads to the stated expression. □

We now prove a corollary regarding μ (defined in (S19)), which provides justification to Eq. 7 in the main text.

Corollary S2. *Let $t_{1/2}$ be the median time for the vertex N population to arise, conditioned on S_1. That is $t_{1/2}$ satisfies*

$$\mathbb{P}(T > t_{1/2} | S_1) = \frac{1}{2}. \tag{S56}$$

Then

$$\lim_{\nu^* \to 0} |t_{1/2} - (\mu - h(z))| = 0 \tag{S57}$$

where

$$h(z) = -\lambda^{-1} \log\left(\frac{\lambda/\alpha}{2^{-1/z}(1 + (\beta/\alpha)^z)^{1/z} - \beta/\alpha} - 1\right). \tag{S58}$$

This also implies $t_{1/2} \sim \mu - h(z)$. Regarding $h(z)$ we have

$$h(1) = 0, \quad h(z) = \lambda^{-1} \log\left(\frac{z\lambda}{\alpha}\right) + O(z)^{-1}, \quad z \to \infty. \tag{S59}$$

Proof. We firstly note, as is apparent from the cumulative distribution function of T (see (S23) for instance), that $t_{1/2}$ is monotone increasing as ν^* decreases. By (S56)

$$\frac{1}{2} = \mathbb{P}(\lambda(T - \mu) > \lambda(t_{1/2} - \mu) | S_1). \tag{S60}$$

Taking the $\nu^* \to 0$ limit we find $\lambda(t_{1/2} - \mu) \to x$, where the convergence is guaranteed as both $t_{1/2}$, μ are monotone increasing. Further, from Corollary S1, the limit x satisfies

$$\frac{[\lambda/\alpha(1+e^x)^{-1} + \beta/\alpha]^z - (\beta/\alpha)^z}{1 - (\beta/\alpha)^z} = \frac{1}{2}. \tag{S61}$$

Solving for x leads to $x = -\lambda h(z)$ as stated. For the asymptotic result we firstly consider

$$d(z) = \frac{\lambda/\alpha}{2^{-1/z}(1 + (\beta/\alpha)^z)^{1/z} - \beta/\alpha} - 2^{\frac{\alpha}{\lambda z}} \tag{S62}$$

Expansions on the numerator and denominator show that $d(z)$ is $O(z^{-2})$. Then

$$-\lambda h(z) = \log(2^{\frac{\alpha}{\lambda z}} - 1 + d(z)) \tag{S63}$$

$$= \log(2^{\frac{\alpha}{\lambda z}} - 1) + \frac{d(z)}{2^{\frac{\alpha}{\lambda z}} - 1} + \dots \tag{S64}$$

As $(2^{\frac{\alpha}{\lambda z}} - 1)^{-1} = O(z)$ we have shown the claimed asymptotic behaviour.

□

S4 Path graph and acyclic graphs

In this section we specialise the results of Sections S2 and S3 firstly to the case of a path graph and then to acyclic graphs.

We first consider a path graph with N vertices and let $\tilde{p}_{1:x} = (1, 2, \dots x)$. In this setting the matrix A from Section S2 has entries

$$a_{ij} = \begin{cases} \lambda(i) & j = i \\ \nu(j,i) & j = i-1 \\ 0 & \text{otherwise,} \end{cases} \tag{S65}$$

and the vector ψ has entries $\psi_i = \alpha(i) + \beta(i) + \nu(i, i+1)$ for $i = 1, \dots, N-1$. We want a useful representation of the sequence $(\Phi_x)_{x=1}^{N-1}$, which we recall is given in terms of the entries of the suitably normalised left and right eigenvectors of A (see (S14)). Because they are easier to work with (i.e. check they indeed are eigenvectors) we first introduce the unnormalised left and right vectors u', v' with entries

$$u'_i = \begin{cases} 1, & i = 1 \\ 0 & 2 \le i \le N-1 \end{cases} \qquad v'_i = \prod_{j=2}^{i} \frac{\nu(j-1, j)}{\lambda - \lambda(j)}, \quad 1 \le i \le N-1 \tag{S66}$$

where the empty product is again set equal to 1. Note for $2 \le x \le N-1$ we have

$$v'_x = \frac{w(\tilde{p}_{1:x})}{\lambda - \lambda(x)}, \tag{S67}$$

here $w(\cdot)$ is again the path weight defined in (S3) . It may be directly verified that these are left and right eigenvectors for A with eigenvalue λ (for v' it is helpful to note that $Av' = \lambda v'$ implies $\nu(i-1, i)v'_{i-1} + \lambda(i)v'_i = \lambda v'_i$) . Now we normalise these, so that they agree with (S14) via

$$\tilde{u} = \frac{\psi \cdot v'}{v'_1} u', \quad \tilde{v} = \frac{1}{\psi \cdot v'} v'. \tag{S68}$$

Our aim is to obtain $\Phi = \tilde{u}_1 \tilde{v}$, and as both $u'_1 = v'_1 = 1$, we see that $\Phi = v'$. Using this, the following corollary demonstrates how the results of the previous section on population growth and time to reach the target simplifies in the path graph setting.

Corollary S3. *The results of Sections S2 and S3 hold with the explicit representations*

$$\Phi_x = \begin{cases} 1 & x = 1 \\ \frac{w(\tilde{p}_{1:x})}{\lambda - \lambda(x)} & 2 \leq x \leq N-1 \end{cases} \tag{S69}$$

$$\nu^* = \nu(N-1, N), \tag{S70}$$

and

$$\mu = \lambda^{-1} \log \left[\lambda^2 \left(\alpha \phi_N \right)^{-1} \right]. \tag{S71}$$

The appearance of ϕ_N in the expression for μ in Corollary (S3) is due to $\phi_N = w(\tilde{p}_{1:N}) = \nu(N-1, N)\Phi_{N-1}$ (to be compared with the general definition of μ (S19)). For referencing, note the definition for the weight of a path p, $w(p)$, and total weight of N, ϕ_N, are in (S3) and (S4). We now move to the case where G is acyclic.

Recall the concept of vertex lineage from the Path distribution section in the main text (which for a chosen cell, is a sequence recording the vertices of cells in the ancestral lineage of our chosen cell). We denote the number of cells at time t with vertex lineage p as $X(p,t)$. The number of cells at a vertex is related to the cells with vertex lineages ending at that vertex via

$$Z_x(t) = \sum_{p \in \mathcal{P}_{1,x}} X(p,t). \tag{S72}$$

For any path p, as above we let the first j terms in p be denoted $p_{1:j}$. The process $(X(p_{1:j}, t))_{j=1}^{l+1}$ is a multitype branching process, and $X(p_{1:j}, t)$ represents the number of cells that have progressed to step j along path p at time t. Births occur to individuals of type i at at rate $\alpha(p_i)$, deaths at rate $\beta(p_i)$ and individuals of type i transition to type $i+1$ at rate $\nu(p_i, p_{i+1})$. Thus we may use our results for path graphs with vertices labelled $1, \ldots, l+1$. Hence via Theorem S1 and Corollary S3 we have the following.

Corollary S4.

$$\lim_{t \to \infty} e^{-\lambda t} X(p,t) = W w(p) \quad a.s. \tag{S73}$$

where $w(p)$ is the path weight given in (S3) *and W is as in Lemma S1.*

Analogously to Corollary S3 we have the following statement regarding the growth of the population at the initial and intermediate vertices.

Corollary S5. *The results of Sections S2 and S3 hold with the explicit representations*

$$\Phi_x = \begin{cases} 1 & x = 1 \\ \frac{1}{\lambda - \lambda(x)} \sum_{p \in \mathcal{P}_{1,x}} w(p) & 2 \leq x \leq N-1 \end{cases} \tag{S74}$$

$$\mu = \lambda^{-1} \log \left(\lambda^2 \left[\alpha \sum_{p \in \mathcal{P}_{1,N}} w(p) \right]^{-1} \right). \tag{S75}$$

where $w(p)$ is the path weight given in (S3).

Note that as the empty product is defined to be 1, $\phi_1 = 1$.

Proof of Corollary S5. Due to Theorem S1 we must demonstrate

$$\lim_{t\to\infty} e^{-\lambda t} Z_x(t) = W \sum_{p\in\mathcal{P}_{1,x}} w(p) \quad a.s. \tag{S76}$$

By (S72) it is enough to show

$$\mathbb{P}\left(\bigcap_{p\in\mathcal{P}_{1,x}} \lim_{t\to\infty} e^{-\lambda t} X(p,t) = W\right) = 1. \tag{S77}$$

Observe that for any events $(A_i)_{i=1}^n$ such that for all i, $\mathbb{P}(A_i) = 1$ then $\mathbb{P}(\cap_i^n A_i) = 1$. Use this with events $A_p = \{\lim_{t\to\infty} e^{-\lambda t} X(p,t) = W\}$ to conclude. □

One particular consequence, by coupling Corollaries S1 and S5 is that for ν^* small

$$\mathbb{P}(T > t|S_1) \approx \frac{\left[\lambda/\alpha(1 + e^{\lambda t}\phi_N \alpha/\lambda^2)^{-1} + \beta/\alpha\right]^z - (\beta/\alpha)^z}{1 - (\beta/\alpha)^z} \tag{S78}$$

which is the conditional version of Eq. 5 in the main text. Differentiating yields an approximation for the conditional density

$$f_{T|T<\infty}(t) \approx \frac{z e^{\lambda t}\phi_N \left(\frac{\lambda/\alpha}{1+e^{\lambda t}\phi_N\alpha/\lambda^2} + \beta/\alpha\right)^{z-1}}{(1 - (\beta/\alpha)^z)\left(1 + \frac{\alpha e^{\lambda t}\phi_N}{\lambda^2}\right)^2}. \tag{S79}$$

S5 Distribution of the path to the target

We continue to take G acyclic. Note that $|\mathcal{P}_{1,N}|$ is finite, and so we let $n = |\mathcal{P}_{1,N}|$. The elements of $\mathcal{P}_{1,N}$ can be enumerated and we use the following notation to do so $\mathcal{P}_{1,N} = \{p^{(1)}, \ldots, p^{(n)}\}$. For each $p^{(i)} \in \mathcal{P}_{1,N}$ we let: the path lengths be $l^{(i)} = |p^{(i)}| - 1$, $u^{(i)} = \nu(p^{(i)}_{l^{(i)}}, N)$ be the final transition rates, and $\eta^{(i)} = w(p^{(i)})/u^{(i)}$. Consider $p^{(i)} \in \mathcal{P}_{1,N}$ and recall that $X(p^{(i)}_{1:j}, t)$ represents the number of cells that have progressed to step j along path i at time t. If we apply the first statement in Theorem S2 to the branching process $(X(p^{(i)}_{1:j}, t)))_{j=1}^{l^{(i)}+1}$ we can deduce that under a time rescaling, the time to traverse path i is approximately exponentially distributed, conditional on the population growth along that path. Therefore the question of which path the target population arises from becomes equivalent to the minimum of a set of exponential random variables. To simplify notation we let,

$$T^{(i)} = T(p^{(i)}) = \inf\{t \geq 0 : X(p^{(i)}, t) > 0\}. \tag{S80}$$

Also, from the Path distribution section of the main text, recall the notation

$$T(\neg p) = \min\{T(q) : q \in \mathcal{P}_{1,N}\backslash\{p\}\}. \tag{S81}$$

We can now prove the precise version of Theorem 2 in the main text.

Theorem S3. *Assume the limits* $\lim_{\nu^*\to 0} \frac{u^{(i)}}{u^{(j)}}$ *exist, but may be infinite, and further that there exists* $i^* \in \{1, \ldots, n\}$ *such that* $\lim_{\nu^*\to 0} \frac{u^{(i)}}{u^{(i^*)}} < \infty$ *for all* $i = 1, \ldots n$. *Then, for* $p \in \mathcal{P}_{1,N}$ *and* $t \in \mathbb{R}$,

$$\lim_{\nu^*\to 0} \mathbb{P}(T(\neg p) - T(p) > t|S_1) = \lim_{\nu^*\to 0} \frac{w(p)}{w(p) + e^{\lambda t}\sum_{q\in\mathcal{P}_{1,N}\backslash\{p\}} w(q)}. \tag{S82}$$

Proof. Consider initially the case when $n = 2$, that is there are only two paths from vertex 1 to vertex N. These two paths must diverge before N. If the population growth along these paths until the target vertex (but excluding the population growth at the target vertex itself) is given, then $T^{(1)}$ and $T^{(2)}$ are the first arrival times in two independent non-homogeneous Poisson processes. Returning to arbitrary n, to use this fact we define the population numbers along all the paths up until the target vertex as

$$\mathcal{X}(s) = (X(p^{(i)}_{1:j}, s))^{n,l^{(i)}}_{i=1,j=1}. \tag{S83}$$

So that we can use this conditional independence, and prior results, we note

$$\mathbb{P}(T(\neg p) - T(p) > t|S_1) = \frac{\mathbb{E}[\mathbb{I}_{S_1}\mathbb{P}(T(\neg p) - T(p) > t|(\mathcal{X}(s))_{s\geq 0})]}{\mathbb{P}(S_1)}. \tag{S84}$$

We now focus on the argument of the above expectation. Let the path under consideration be path 1 ($p = p^{(1)}$) and

$$\kappa^{(i)} = \lim_{\nu^*\to 0} \frac{u^{(i)}}{u^{(i^*)}}. \tag{S85}$$

We know, by Theorem S2, that each $T^{(i)}$ appropriately centred converges. If we centre each $T^{(i)}$ by the same quantity, then the ordering is preserved. That is, clearly $T^{(1)} < T^{(2)} \iff T^{(1)} - \mu^{(i)} < T^{(2)} - \mu^{(i)}$ for any $\mu^{(i)}$. As $T^{(i)}$ is the target time along the path graph $p^{(i)}$, Corollary S3 gives the appropriate centring. In particular with

$$\mu^{(i)} = \lambda^{-1} \log(\lambda^2[\alpha\eta^{(i)}u^{(i)}]^{-1}) \tag{S86}$$

we have, by the first statement of Theorem S2 and Corollary S3,

$$\lim_{\nu^*\to 0} \mathbb{P}(\lambda(T^{(i)} - \mu^{(i)}) > x|(\mathcal{X}(s))_{s\geq 0}) = \exp(-e^x W\lambda/\alpha)\, a.s. \tag{S87}$$

For order preservation (of the $T^{(i)}$s) we take $\mu^{(i^*)}$ as a common centring of all $T^{(i)}$, and use $T^{(i)} - \mu^{(i^*)} = T^{(i)} - \mu^{(i)} + \mu^{(i)} - \mu^{(i^*)}$ and

$$\lim_{\nu^*\to 0} \mu^{(i)} - \mu^{(i^*)} = \lambda^{-1} \log\left(\frac{\eta^{(i^*)}}{\eta^{(i)}\kappa^{(i)}}\right). \tag{S88}$$

Note the above expression may be $-\infty$. Using this, and exponentiating (which again preserves order), leads to

$$\lim_{\nu^*\to 0} \mathbb{P}(\exp[\lambda(T^{(i)} - \mu^{(i^*)})] > t|(\mathcal{X}(s))_{s\geq 0}) = \exp\left(-\frac{tW\lambda\eta^{(i)}\kappa^{(i)}}{\alpha\eta^{(i^*)}}\right)\ a.s. \tag{S89}$$

At this point we note that if $\kappa^{(i)} = 0$, then for fixed $(\mathcal{X}(s))_{s\geq 0}$, the random variables $\exp[\lambda(T^{(i)} - \mu^{(i^*)})]$ converges to $+\infty$ almost surely. Recall the $T^{(i)}$ are conditionally independent, and hence convergence of the marginal distributions implies joint convergence. Therefore for $(x_j)^n_{j=1}$ with each $x_j \geq 0$, almost surely we have

$$\lim_{\nu^*\to 0} \mathbb{P}\left[\left(\exp[\lambda(T^{(j)} - \mu^{(i^*)})] > x_j\right)^n_{j=1} |(\mathcal{X}(s))_{s\geq 0}\right] = \prod_{j=1}^n \exp\left(-\frac{x_j W\kappa^{(j)}\eta^{(j)}\lambda}{\eta^{(i^*)}\alpha}\right). \tag{S90}$$

Including the shift t, we have for fixed $(\mathcal{X}(s))_{s\geq 0}$ the random vector $(\exp[\lambda(T^{(1)} + t - \mu^{(i^*)})], \exp[\lambda(T^{(j)} - \mu^{(i^*)})])^n_{j=2}$ jointly tends in distribution to the random vector $(U_i)^n_{i=1}$. Note that only $T^{(1)}$ is shifted, due to the path under consideration being $p^{(1)}$. If $W = 0$, all the $U_i = +\infty$ a.s., instead if $W > 0$ each U_i is independent and for $2 \leq i \leq n$ has distribution

$$U_i \stackrel{d}{=} \begin{cases} +\infty \text{ if } \kappa^{(i)} = 0 \\ E_i \text{ if } 0 < \kappa^{(i)} < \infty \end{cases} \tag{S91}$$

where E_i is an exponential random variable with parameter $\frac{W\kappa^{(i)}\eta^{(i)}\lambda}{\eta^{(i^*)}\alpha}$. For $i = 1$, instead we have $U_1 \overset{d}{=} +\infty$ if $\kappa^{(1)} = 0$, else U_1 is distributed as an exponential with parameter $\frac{e^{-\lambda t}W\kappa^{(1)}\eta^{(1)}\lambda}{\eta^{(i^*)}\alpha}$. As, up to null events, $\{W > 0\} = S_1$, and using standard results for the ordering of exponential variables (looking at the minimum of the U_i), we have

$$\lim_{\nu^*\to 0} \mathbb{I}_{S_1}\mathbb{P}(T(p) + t < T(\neg p)|(\mathcal{X}(s))_{s\geq 0}) = \mathbb{I}_{S_1}\frac{\kappa^{(1)}\eta^{(1)}}{\kappa^{(1)}\eta^{(1)} + e^{\lambda t}\sum_{j=2}^{n}\kappa^{(j)}\eta^{(j)}}. \tag{S92}$$

The right hand side of (S92) is an element of $[0, 1]$. 0 is obtained when $\kappa^{(1)} = 0$ (the final transition rate along the path is small, in the sense of the limit (S85), relative to other paths) and 1 is obtained if $\kappa^{(i)} = \delta_{1,i}$ (the final transition rate along the path is the largest relative to other paths). Coupling the above with (S84) gives the result. □

S6 Cyclic graphs

Suppose G is as before, but without the acyclic assumption. For cyclic graphs in place of $\mathcal{P}_{1,N}$ we must consider $\mathcal{W}_{1,N}$, which we define to be the set of walks between the root and vertex N. The definition of vertex lineages holds identically for walks. Therefore for $\omega \in \mathcal{W}_{1,N}$ we let

$$T(\omega) = \inf\{t \geq 0 : X(\omega, t) > 0\} \tag{S93}$$

where $X(\omega, t)$ is as given in the Path distribution section of the main text. Here we show that so long as the number of transitions between vertices is finite, which corresponds to finitely many back transitions, we may map a graph containing cycles to an acyclic one and then use the results given above.

The set of walks between the root and the target vertex of length at most $i - 1$ will be denoted

$$\mathcal{W}_{1,N}^{(i)} = \{\omega \in \mathcal{W}_{1,N} : |\omega| \leq i\}. \tag{S94}$$

Further for a graph $G = (V, E)$, let the vertex parameters be the birth and death rates associated to the vertices of G and the edge parameters the transition rates associated with the edges.

Proposition S2. *For a given graph $G = (V, E)$ with associated vertex and edge parameters, and $i \geq 1$ which is the upper-length of walks that we consider on G, we can construct an acyclic graph $G' = (V', E')$ (dependent on i), which coupled with appropriate vertex and edge parameters, permits a bijection $g : \mathcal{W}_{1,N}^{(i)} \mapsto \mathcal{P}'_{1,|V'|}$. This bijection possesses the property that for $\omega \in \mathcal{W}_{1,N}^{(i)}$*

$$T(\omega) \overset{d}{=} T'(g(\omega)) \tag{S95}$$

where $T'(g(\omega))$ is defined as in (S93) *but with the process on G' with its associated parameters.*

Before giving the proof we briefly demonstrate this on the graphs shown in Fig. A. This elucidates the general idea.

Here

$$\mathcal{W}_{1,N}^{(4)} = \{(1, 2, 4), (1, 2, 3, 4), (1, 3, 4), (1, 3, 2, 4)\}. \tag{S96}$$

It is clear these may be mapped to the paths in G'

$$\mathcal{P}'_{1,|V'|} = \{(1, 2, 6), (1, 2, 3, 6), (1, 4, 6), (1, 4, 5, 6)\}. \tag{S97}$$

Further $T((1, 2, 4)) \overset{d}{=} T'((1, 2, 6))$ so long as the transition rates associated with edges $(1, 2)$ and $(2, 4)$ on G are equal to the rates with $(1, 2)$ and $(2, 6)$ on G' and that the birth and death rates at vertices 1 and 2 are equal in both cases.

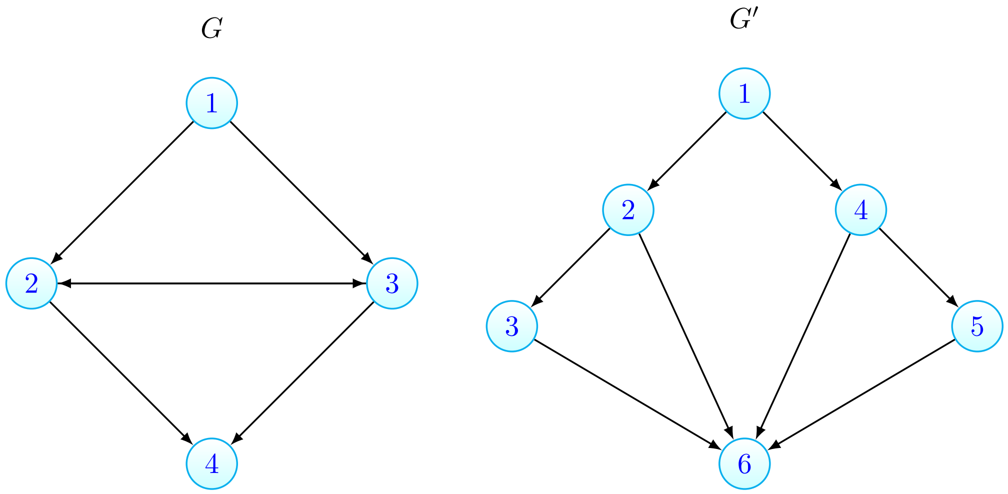

Fig A. Mapping cyclic graphs to acyclic graphs

Proof of Proposition S2. To avoid cumbersome notation and as the idea is illustrated in Fig. A we sketch an algorithm. We enumerate the set $\mathcal{W}^{(i)}_{1,N} = \{\omega^{(1)}, \omega^{(2)}, \dots \omega^{(\mathcal{M})}\}$, where $\mathcal{M} = |\mathcal{W}^{(i)}_{1,N}|$. Again we use the notation $l^{(j)}$ for the length of the jth walk, that is $l^{(j)} = |\omega^{(j)}| - 1$. The graph G' is constructed iteratively. Let $V'_1 = \{1, 2, \dots, l^{(1)}, N'\}$, where N' is a placeholder and will ultimately be $|V'|$. Similarly $E'_1 = \{(1,2), \dots, (l^{(1)}, N')\}$ and $p^{(1)} = (1, 2, \dots, l^{(1)}, N')$.

Then for each further $\omega^{(j)}$, $2 \le j \le \mathcal{M}$, from the previously considered walks select the walk that is identical to $\omega^{(j)}$ for the greatest number of vertices, say $\omega^{(k)}$, $1 \le k < j$. If $\omega^{(j)}$ and $\omega^{(k)}$ agree up until their mth element, and $l^{(j)} - m > 0$ then add $l^{(j)} - m$ elements to V'_{j-1} (new vertices starting with $|V'_{j-1}|$). This creates V'_j. Denote the new vertices as $(v^{(j)}_x)^{l^{(j)}-m}_{x=1}$. As $\omega^{(k)}$ has previously been considered it will have an associated path $p^{(k)}$, whose final element we will require to construct E_j: to E_{j-1} add the edges $(p^{(k)}_m, v^{(j)}_1)$, $\{(v^{(j)}_x, v^{(j)}_{x+1})\}^{l^{(j)}-m-1}_{x=1}$ and $(v^{(j)}_{l^{(j)}-m}, N')$. This creates E_j. For $p^{(j)}$ we let $p^{(j)}_{1:m} = p^{(k)}_{1:m}$ and then add the new vertices of V'_j, and terminate the path at N'. If $l^{(j)} = m$ (and so the walk $\omega^{(j)}$ agrees with $\omega^{(k)}$ until its penultimate element) let $V'_j = V'_{j-1}$ and add the edge $(p^{(k)}_m, N')$ to E'_{j-1}. Then $p^{(j)}$ is equal to $p^{(k)}_{1:m}$ concatenated with N'. Finally our constructed graph is $G' = (V', E') = (V'_{\mathcal{M}}, E'_{\mathcal{M}})$ with $N' = |V'|$.

Identifying $g(\omega^{(j)}) = p^{(j)}$, we see that if $\omega^{(j)}$, $\omega^{(k)}$ agree until their mth element then so also will $g(\omega^{(j)})$, $g(\omega^{(k)})$, but after this the paths diverge. We further require that the birth and death rates for the population type represented by the kth element of ω should be the same as the kth element of $g(\omega)$. Similarly for the transition rates between the kth and $k+1$th element of ω and $g(\omega)$.

$\square$

Coupling the above proposition with the results of Section S4 allows one to characterise

$$\min_{\omega \in \mathcal{W}^{(k)}_{1,N}} T(\omega) \tag{S98}$$

in terms of the weights of the walks. Further for a set of walks $\mathcal{W}^{(i)}_{1,N}$, Theorem S3 holds but now with the walk weights, defined analogously to the definition given in (S3). Note that the Proposition S2 holds regardless of whether the root is a source vertex, or whether the vertex 1 population is the most fit. However,

if transitions are permitted into the root vertex, then for any given walk, there is no guarantee that the cells associated with the first element of that walk are the most fit. This would leave us unable to apply the results derived in earlier sections.

While the above proposition allows us to consider back transitions, the following result demonstrates that when the transition rates between vertices are small, the first initiation of the target is dominated by the paths, as opposed to walks containing cycles. This offers a secondary justification for mainly focusing on acyclic graphs. To state the result let us define $q : \mathcal{W}_{1,N} \mapsto \mathcal{P}_{1,N}$ as the operation reducing walks to their respective paths. This may be accomplished recursively by searching for the first cycle in ω and replacing this with the first element in the cycle. Then repeating the same procedure on the reduced walk until it is a path. This operation is the same as that which maps trajectories to their corresponding trajectory class in [6]. Further, for $p \in \mathcal{P}_{1,N}$ let

$$\mathcal{W}^{(i)}[p] = \{\omega \in \mathcal{W}^{(i)}_{1,N} : q[\omega] = p, \omega \neq p\} \tag{S99}$$

that is the walks of length at most $i - 1$ which when reduced to paths are p, but excluding p itself. Then with

$$\nu_{\max} = \max\{\nu(i,j) : (i,j) \in E\} \tag{S100}$$

we have the following.

Proposition S3. *For $i \geq 1$, $p \in \mathcal{P}_{1,N}$ such that $\mathcal{W}^{(i)}[p]$ is non-empty, and all $\omega \in \mathcal{W}^{(i)}[p]$*

$$\lim_{\nu_{\max}\to 0} \lim_{\nu^*\to 0} \mathbb{P}(T(p) < T(\omega)|S_1) = 1. \tag{S101}$$

Proof. We construct an acyclic graph with appropriate vertex and edge parameters as in Proposition S2 and use the mapping g from there with domain $\mathcal{W}^{(i)}_{1,N}$. As before let $\mathcal{P}'_{1,|V'|}$ be the codomain of g. Then for path $p \in \mathcal{W}^{(i)}_{1,N}$ and walk $\omega \in \mathcal{W}^{(i)}[p]$, $g(p)$, $g(\omega) \in \mathcal{P}'_{1,|V'|}$. Note due to the manner in which the edge parameters are chosen $\{\nu(i,N) : i \in \mathcal{N}^-(N)\} = \{\nu(i,|V'|) : i \in \mathcal{N}^-(|V'|)\}$ and hence ν^* remains unchanged. Similarly $\nu_{\max}$ is unchanged. Therefore,

$$\lim_{\nu^*\to 0} \mathbb{P}(T(p) < T(\omega)|S_1) = \lim_{\nu^*\to 0} \mathbb{P}(T'(g(p)) < T'(g(\omega))|S_1). \tag{S102}$$

Furthermore each vertex and edge in p is also present in ω. Hence the weight of $g(\omega)$ satisfies $w(g(\omega)) = w(g(p))\tilde{w}(g(\omega))$ where $\tilde{w}(g(\omega))$ is the product of transition rates along the edges existing in cycles in ω divided by the costs of vertices in the cycles. Then by Theorem 5

$$\lim_{\nu^*\to 0} \mathbb{P}(T'(g(p)) < T'(g(\omega))|S_1) = \frac{1}{1 + \tilde{w}(g(\omega))}. \tag{S103}$$

As $\tilde{w}(g(\omega))$ contains transition rates, this leads to the stated result upon taking $\nu_{\max} \to 0$. □

S7 Simulation for alternative transition formulation

See Fig. B for a comparison of simulations with transitions following $(x) \to (y)$ with the hitting time results derived in the Results section of the main text.

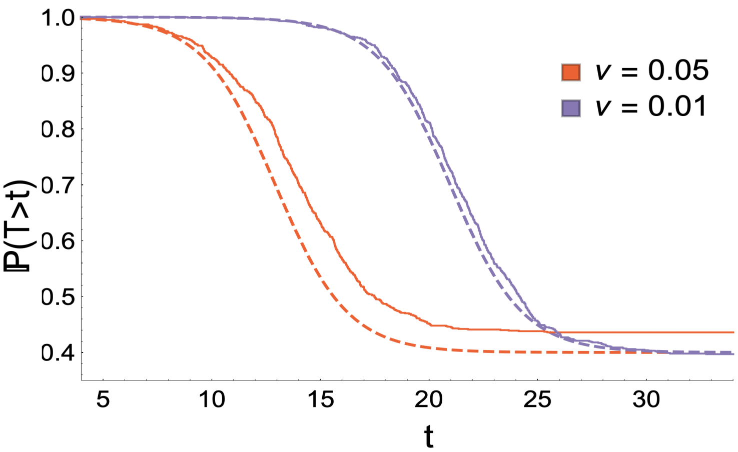

Fig B. Alternative transition formulation: Here we compare analogous simulations to Fig 3b in the main text, except that transitions follow $(x) \to (y)$, with the theoretical approximation of Eq. 5 in the main text. Dashed lines give the theoretical result, joined lines represents the empirical distribution obtained from 1000 realisations. Despite Eq. 5 of the main text begin derived under the formulation when transitions follow $(x) \to (x), (y)$, there is excellent agreement between the simulations and theory. Parameters: $\alpha = \alpha(2) = \alpha(3) = \alpha(4) = 1, \beta = 0.4, \beta(2) = \beta(3) = 1.3, \beta(4) = 0.6, z = 1$; legend displays value of $\nu = \nu(1,2) = \nu(2,3) = \nu(3,4)$ used.

S8 Derivation of select formulas in the Imperfect drug penetration: combination therapy section

Here we outline the derivation of the expressions contained in the Imperfect drug penetration: combination therapy section of the main text, in particular formulas 16-18. These expressions were derived with the assistance of the Mathemetica software [8].

We start with Eq. 16. Let the set of stepwise paths be $\mathcal{P}_{\mathrm{SE}}$ and the set of directed paths be $\mathcal{P}_{\mathrm{DE}}$. For a path $p = (p_1, \ldots p_{l+1})$ with number of edges (path length) l, write $c(p) = \prod_{i=2}^{l}(\lambda - \lambda(p_i))$. Note for any path considered here, if we follow the path on Fig. 7a, then $c(p)$ is the product of the vertex labels, taken over all intermediate vertices (excluding the root and the target). Further, let us introduce $r = n_{\mathrm{D}}/n_{\mathrm{S}}$ and $f = (n_{\mathrm{D}} + n_{\mathrm{S}})/n_{\mathrm{Tot}}$. This implies $n_{\mathrm{DD}} = (1-f)n_{\mathrm{Tot}}$, $n_{\mathrm{S}} = f n_{\mathrm{Tot}}/(1+r)$, $n_{\mathrm{D}} = r f n_{\mathrm{Tot}}/(1+r)$. Then

$$\phi_N = \frac{n_{\mathrm{D}}}{n_{\mathrm{D}} + n_{\mathrm{DD}}} \frac{n_{\mathrm{DD}}}{n_{\mathrm{S}} + n_{\mathrm{DD}}} m^2 \nu^2 \sum_{p \in \mathcal{P}_{\mathrm{SE}}} (c(p))^{-1} + \frac{n_{\mathrm{DD}}}{n_{\mathrm{DD}} + n_{\mathrm{D}}} \nu^2 m \sum_{p \in \mathcal{P}_{\mathrm{DE}}} (c(p))^{-1} \tag{S104}$$

$$= \left[1 + \frac{(1-f)(1+r)}{rf}\right]^{-1} \left[1 + \frac{f}{(1+r)(1-f)}\right]^{-1} m^2 \nu^2 \sum_{p \in \mathcal{P}_{\mathrm{SE}}} (c(p))^{-1} + \tag{S105}$$

$$\left[1 + \frac{r}{(1+r)(1-f)}\right]^{-1} \nu^2 m \sum_{p \in \mathcal{P}_{\mathrm{DE}}} (c(p))^{-1}. \tag{S106}$$

Using the approximation of Eq. 7 in the main text, we have $t_{1/2}(r) = -\lambda^{-1} \log(\lambda^{-1} \phi_N z))$. Also recall $z = \gamma n_s = \gamma f n_{\mathrm{Tot}}/(1+r)$. Differentiating $t_{1/2}(r)$ and evaluating at $r = 0$ leads to

$$\frac{d}{dr} t_{1/2}(0) = \frac{\frac{1}{1-f} - \frac{fm(d+s)^3}{ds(d^2+3ds+s^2)}}{\lambda}. \tag{S107}$$

Increasing the relative size of the single drug compartment speeds up resistance when $\frac{d}{dr}t_{1/2}(0) < 0$ or equivalently when

$$\frac{ds(d^2+3ds+s^2)}{1-f} > fm(d+s)^2. \tag{S108}$$

Expansions in f and s/d (recall $f \ll 1, s/d \ll 1$), keeping only leading order terms and using the definition of f leads to Eq. 16 in the main text.

Turning to Eq. 17 of the main text, we wish to find r such that $\frac{d}{dr}t_{1/2}(r) = 0$. The numerator of $\frac{d}{dr}t_{1/2}(r)$ is quadratic in r. After solving for both roots and expanding in powers of s/d, we see the leading term for one of the roots is -1. Thus the root of interest is the other root, which to leading order is the expression in Eq. 17.

For Eq. 18 of the main text, we use our main result of Theorem 2 in the main text. With ϕ_N as given above in (S104), this yields

$$\mathbb{P}(T^{\mathrm{SE}} < T^{\mathrm{DE}}) \approx \frac{\frac{n_{\mathrm{D}}}{n_{\mathrm{D}}+n_{\mathrm{DD}}}\frac{n_{\mathrm{DD}}}{n_{\mathrm{S}}+n_{\mathrm{DD}}}m^2\nu^2\sum_{p\in\mathcal{P}_{\mathrm{SE}}}(c(p))^{-1}}{\phi_N} \tag{S109}$$

$$= \left(1+\frac{(n_{\mathrm{DD}}+n_{\mathrm{S}})}{n_{\mathrm{D}}m}\frac{d^3s+3s^2d^2+ds^3}{(d+s)^3}\right)^{-1}. \tag{S110}$$

Upon expanding in s/d, to leading order we have Eq. 18.

References

1. K. B. Athreya and P. E. Ney, *Branching Processes*, Dover Publications, 2004.

2. T. H. Cormen, C. E. Leiserson, R. L. Rivest, and C. Stein, *Introduction to Algorithms, Third Edition*, vol. 7, The MIT Press, Cambridge, Massachusetts., third ed., 2009.

3. R. Durrett, *Branching Process Models of Cancer*, Stochastics in Biological Systems, Springer, 2015.

4. Y. Iwasa, F. Michor, and M. A. Nowak, *Evolutionary dynamics of invasion and escape*, Journal of Theoretical Biology, 226 (2004), pp. 205–214.

5. S. Janson, *Functional limit theorems for multitype branching processes and generalized Pólya urns*, Stochastic Processes and their Applications, 110 (2004), pp. 177–245.

6. A. C. Tadrowski, M. R. Evans, and B. Waclaw, *Phenotypic Switching Can Speed up Microbial Evolution*, Scientific Reports, 8 (2018), p. 8941.

7. R. S. Varga, *Matrix Iterative Analysis*, Prentice-Hall International, 1962.

8. Wolfram Research, Inc., *Mathematica, Version 11.3*. Champaign, IL, 2018.
